# Examining the experimental effects of concentration and temperature on the viscosity of nanofluid containing graphene oxide, suggesting a correlation, and developing a neural network

**DOI:** 10.1038/s41598-026-48249-0

**Published:** 2026-04-15

**Authors:** Reza Aghayari, Bahram Keyvani, Davood Toghraie, Soheil Salahshour

**Affiliations:** 1 Department of Chemistry, Sav.C., Islamic Azad University, Saveh, Iran; 2 Department of Chemistry, Sav.C., Islamic Azad University, Saveh, Iran; 3 Department of Mechanical Engineering, Kho.C., Islamic Azad University, Khomeinishahr, Iran; 4 Efficiency and Smartization of Energy Systems Research Center, Kho.C., Islamic Azad University, Khomeinishahr, Iran; 5 Faculty of Engineering and Natural Sciences, Istanbul Okan University, Istanbul, Turkey; 6 Faculty of Engineering and Natural Sciences, Bahcesehir University, Istanbul, Turkey; 7 Research Center of Applied Mathematics, Khazar University, Baku, Azerbaijan; 8 Faculty of Science and Letters, Piri Reis University, Tuzla, Istanbul, Turkey

**Keywords:** Viscosity, Graphene oxide nanofluid, Correlation coefficient, Mean squared error, Engineering, Materials science, Mathematics and computing, Nanoscience and technology, Physics

## Abstract

The application of nanotechnology represents a recent initiative to explore thermal sciences across various sectors. With substantial investments from developed nations in nanotechnology, extensive research has been conducted on the thermophysical properties of nanofluids, including viscosity and thermal conductivity. The present study measured the viscosity of Graphene oxide/water nanofluids at concentrations ranging from 5 to 8 g/L and temperatures between 20 °C and 35 °C. The objective was to establish an empirical relationship using Origin software to encompass all data with minimal error and a maximum correlation coefficient. The findings revealed that as the concentration of nanoparticles in the base fluid increased, the viscosity correspondingly rose. Conversely, at elevated temperatures, viscosity decreased. Furthermore, the viscosity of the Graphene oxide nanofluid was modeled using a Perceptron Artificial Neural Network with a 2-7-1 architecture, with concentration and temperature as the two inputs and viscosity as the output. The results for the proposed empirical relationship and the artificial neural network demonstrated that the mean squared error, mean absolute error, root mean squared error, and correlation coefficient were 0.002188, 0.0172564, 0.04677, and 0.98704, respectively, indicating a successful evaluation. The maximum positive and negative errors for the acquired data were 0.0375% and 0.068%, respectively. The values obtained were − 7.71% < Margin of Deviation (Correlation) < + 7.5% and − 9.55% < Margin of Deviation (Artificial Neural Network) < + 7.7%.

## Introduction

Common fluids used for heat transfer and energy transport in industry generally include Water and other fluids. With increasing competition in various industries and the role of energy in production costs, these industries are rapidly advancing towards the development of advanced fluids with high thermal indices. Jin et al.^1^ employed an artificial neural network model to investigate the rheological properties of hybrid non-Newtonian ferrofluids composed of Fe-CuO, Water, and ethylene glycol. The results showed that the Group Method of Data Handling method outperformed other regression models, achieving a Coefficient of Determination (R^2^) of 0.99436 and a Root Mean Square Error (RMSE) of 2.0135 for the viscosity function. Finally, optimization using the NSGA II algorithm determined the best parameter values, confirming the high efficiency of these methods in modeling the rheological behavior of nanofluids. Behrozifard et al.^2^ studied how various nanofluids influence heat transfer enhancement in a plate heat exchanger. They examined GO/Water and AL_2_O_3_–/water hybrid nanofluids at weight concentrations of 0.01%, 0.02%, and 0.03%. The results showed that the highest thermal efficiency was achieved at a 0.03% concentration, with GO exhibiting the greatest impact, improving heat exchanger performance by 15.94%. The hybrid and water nanofluids showed improvements of 11.86% and 7.4%, respectively.Sunder et al.^3^ synthesized magnetic manganese oxide (Mn₃O₄) nanowires using the solvothermal method and characterized them via X-ray diffraction, X-ray photoelectron spectroscopy, and vibrating sample magnetometry. Stable Mn₃O₄/ethylene glycol nanofluids with particle volume fractions from 0 to 1.25% were prepared, and their thermal conductivity and viscosity were measured at temperatures ranging from 20 to 60 °C and magnetic fields from 0 to 2350 Gauss. The results showed that at 1.25% volume fraction, thermal conductivity increased by 18.8% at 60 °C, while viscosity increased by 89.4% at 20 °C compared to the base fluid. The ANFIS algorithm successfully predicted the target data, with root-mean-square errors of 0.0013744 and 0.311183 for thermal conductivity and viscosity, respectively. Esfe et al.^4^ studied the thermophysical properties of the MWCNT–ZnO (30–70%)/(Society of Automotive Engineers oil) SAE (40 hybrid nanofluid at different solid volume fractions, temperatures, and shear rates. Using a knowledge management cycle and Response Surface Methodology (RSM), they developed several viscosity models, with the fifth-order model achieving the highest accuracy. The R^2^, predicted R^2^, and adjusted R^2^ were all 0.9998, confirming the model’s excellent precision. Evaluation using deviation metrics and matching graphs indicated that the Margin of Deviation (MOD) remained within ± 2%. Through knowledge management, the optimal viscosity was determined to be 209.53 mPa·s at 31.156 °C, a volume fraction of 0.063%, and a shear rate of 933.923 s^−1^. Rostami et al.^5^ investigated the viscosity of Graphene oxide and copper oxide in a base fluid consisting of a water-ethylene glycol mixture. Their proposed equation showed a good agreement with experimental values, with a correlation coefficient of 0.9995 and an error tolerance within ± 0.4%. Additionally, data were optimized using neural networks, and correlations were provided. The thermal conductivity of this nanofluid increased by approximately 20% at a 1% volume fraction and 40 °C, and by 27% at 60 °C. Esfe et al.^6^ evaluated the thermal conductivity and viscosity of carbon nanotube nanofluids in oil at volume fractions ranging from 0.1% to 1% using response surface methodology and artificial neural networks. Their results indicated an increase in viscosity with increasing volume fraction, with correlation coefficients of 0.9964, 0.9999, and 0.9980 for thermal conductivity and viscosity, respectively, demonstrating the accuracy of these methods against experimental data. Dong et al.^7^ prepared Graphene/propylene glycol nanofluids with a 60% weight fraction as the base fluid and assessed their thermal conductivity and viscosity. The results showed that the thermal conductivity of nanofluids increases with higher Graphene concentration and temperature, while viscosity increases with Graphene concentration and decreases significantly with temperature. When the volume fraction of nanographene exceeds 0.2% by weight, the increase in thermal conductivity due to higher Graphene mass concentration significantly diminishes. In contrast, viscosity increases substantially, with no notable improvement in heat transfer enhancement. Another study examined the impact of temperature (T) and volume fraction of nanoparticles (φ) on the viscosity of hybrid silica-alumina-MWCNT/water nanofluids. The results showed that the viscosity of hybrid silica-alumina-MWCNT/water nanofluids increases with φ. Numerically, the viscosity increased from 1.55 to 3.26% at T = 40 °C. The results indicate that the viscosity of nanofluids changes more at lower temperatures and higher φ values^8^. Toghraie et al.^9^ examined the dynamic viscosity of Ag/ethylene glycol nanofluids over a temperature range of 25 to 55 °C and volume fractions from 0.2 to 2%. Their results indicated that artificial neural networks can predict the viscosity of silver/ethylene glycol nanofluids with higher accuracy than empirical relationships, achieving a mean squared error (MSE) of 0.0012 and a maximum error of 0.0858. In another study conducted by Xu et al.^10^, the rheological behavior of Graphene oxide/water nanofluids was examined at different mass fractions, including 1.0, 1.5, 2.0, 2.5, and 3.5 mg/mL, across various temperature ranges (25 to 50 °C) and several shear rates (12.23 to 122.3 s^−1^). The nanofluid’s stability was assessed using zeta potential measurements. The results indicated that the nanofluid exhibits non-Newtonian behavior. This trend is similar to the power-law model. Finally, an artificial neural network was developed to predict the viscosity of the nanofluid at different mass fractions and temperatures, achieving an R^2^ value of 0.99. Ranjbarzadeh et al.^11^ investigated the effects of various parameters on heat transfer using Graphene oxide nanofluids in turbulent flow. The thermal conductivity and viscosity of the nanofluids were investigated at temperatures ranging from 25 to 75 °C and volume fractions of 0 to 0.15%. The optimal increases in thermal conductivity and viscosity were found to be 11.2% at 75 °C and 0.15% volume fraction, respectively. The heat transfer coefficient of the nanofluid was 34.7% higher than that of the base fluid. Gao et al.^12^ used a radial basis function neural network to model the dynamic viscosity of a MgO/SAE 5W30 oil hybrid nano-lubricant. Their results showed that increasing the temperature from 5 to 55 $\:^{\circ}$C decreased viscosity, and increasing the shear rate from 50 to 1000 resulted in a viscosity reduction from 400 to 25 centipoise. Salimi et al.^13^ used a combination of graphene/copper oxide/alumina nanofluids with an ethylene glycol-water mixture to measure thermal conductivity. The results were evaluated using an Artificial Neural Network with 78 data points. Validation of the experimental data with the ANN, which achieved an R^2^ of 0.99496, was confirmed. Sahin et al.^14^ modeled the data obtained for the viscosity of iron oxide NF in the concentration range of 0.1 to 1% by mass and a temperature range of 20 to 60 °C using an ANN. The neural network results were in good agreement with the experimental data, with an R^2^ of 0.99968 and an MSE of 4.51E-06. Zakeri et al.^15^ investigated a water-based graphene oxide nanofluid in a counter-current double-pipe heat exchanger. They found that increasing the nanoparticle concentration and flow rate enhanced the heat transfer coefficient, and the nanofluid outperformed the base fluid. Additionally, radial basis function and multi-layer perceptron models were developed to predict the heat transfer coefficient, with the radial basis function model showing higher accuracy. Kanti et al.^16^ investigated the effect of incorporating a hybrid nanofluid (HNF) composed of graphene oxide (GO) and MXene (in a 90:10 ratio) into the vanadium electrolyte for vanadium redox flow batteries (VRFBs). The primary objective was to enhance battery performance by augmenting the nanofluid’s physical and electrical properties. The stability, rheological behavior (Newtonian/non-Newtonian), thermal conductivity (TC), and electrical conductivity (EC) of the HNF were evaluated at varying weight concentrations and temperatures ranging from 10 to 45 °C. The results indicated that the HNF remained stable under these conditions and exhibited Newtonian behavior. Notably, at 45 °C and a concentration of 0.1 wt%, the electrical conductivity (EC) increased by up to 20.5% and the thermal conductivity (TC) by up to 6.81% compared to the base vanadium electrolyte. Subsequently, an advanced explainable machine learning approach, LSBoost, was employed, utilizing 5-fold cross-validation to prevent overfitting, to develop a model for predicting the properties of HNF (TC, EC, and viscosity-VST). Model parameter optimization was performed using the Bayesian technique. The resulting models demonstrated very high predictive accuracy, with coefficients of determination (R^2^) for TC, EC, and VST of 0.9981, 0.99, and 0.9954, respectively, and negligible prediction errors (RMSE and MAE).

Kanti et al.^17^ investigated the dispersion and stability of alumina (Al_2_O_3_) and graphene oxide (GO) nanoparticles in water, as well as the thermal and physical properties of their hybrid nanofluid (HNF) at different mixing ratios. Properties such as thermal conductivity (TC) and viscosity (VST) were measured at volume concentrations of 0.1-1% and temperatures of 30–60 °C. The results indicated that at 60 °C and a 1% volume concentration, the thermal conductivity of the GO nanofluid increased by 43.9% compared to the Al_2_O_3_ nanofluid. Furthermore, increasing the GO content in the hybrid nanofluid enhanced both its thermal conductivity and viscosity. Regression equations were developed to estimate the thermal conductivity and viscosity of the hybrid nanofluids. Finally, two advanced machine learning methods (a Bayesian-optimized support vector machine and a deep neural network) were employed to accurately predict the thermophysical properties of these nanofluids, achieving accuracies of 97.15%-99.91%.Kanti et al.^18^ focused on producing single-component and hybrid water-based nanofluids using graphene oxide (GO) and coal fly ash (CFA) nanoparticles at mixing ratios of 50:50 and 30:70. To enhance the stability of the nanofluids, the surfactant PVP was utilized. The thermal conductivity (TC) and viscosity (VST) properties of the nanofluids were investigated across a concentration range of 0.1 to 1.0 vol% and temperatures from 30 to 60 °C. At a 1% vol concentration and 30 °C, the GO nanofluid exhibited the highest viscosity enhancement (177%), while the GO/CFA (50:50) and GO/CFA (30:70) mixtures showed increases of 152% and 113%, respectively. Regression equations were developed to predict the TC and VST of the hybrid nanofluids. The findings indicated that GO nanofluid and HNF with a 30:70 ratio are suitable for thermal applications across all temperatures, whereas HNF with a 50:50 ratio is beneficial at 45 °C and above.

Table 1 presents the proposed relations for the viscosity of nano fluid provided by various researchers. The novelty of the present study lies not only in introducing a new empirical correlation obtained by curve-fitting the experimental viscosity data using Origin software, but also in integrating this correlation with an artificial neural network (ANN) model for predictive validation. In contrast to previous studies, this work systematically investigates the effects of temperature (20–35 °C) and nanoparticle concentration (5–8 g/L), providing a more comprehensive evaluation of the behavior of graphene oxide/water nanofluids within these specific ranges. Additionally, a detailed uncertainty analysis was performed to enhance the reliability of the experimental measurements and to verify the robustness of both the empirical and ANN models. The high accuracy demonstrated by the proposed correlation and ANN models, reflected in their low error metrics (e.g., MSE) and high R^2^ values, confirms their ability to accurately capture the nonlinear viscosity behavior of the nanofluid. Overall, this study presents a combined experimental, analytical, and predictive framework that offers practical applicability and scientific value, providing insights not reported in previous investigations of GO-based nanofluids.

**Table 1:** Some empirical relations proposed in various studies.

Reference	Proposed relations presented
Irani et al.^19^	$\:\mu\:=233.2713{\phi\:}^{0.8623}{\left(\frac{1}{T}\right)}^{0.8623}-2.6698{\phi\:}^{0.4821}+0.9145$
Tian et al.^20^	$\:\frac{{\mu\:}_{nf}}{{\mu\:}_{bf}}=0.50013+0.019722\left(T\right)+4.23872\left(\phi\:\right)-0.052336\left(T\phi\:\right)$
Esfe et al.^21^	$\:{\mu\:}_{nf}=1+32.7{\phi\:}_{p}-7214{\phi\:}_{p}^{2}+714600{\phi\:}_{p}^{3}-0.194*{10}^{8}{\phi\:}_{p}^{4}){\mu\:}_{f}$
Ajeena et al.^22^	$\:\frac{{\mu\:}_{nf}}{{\mu\:}_{bf}}=0.99761+0.26995{\phi\:}_{p}^{0.32737}-0.03587{T}^{0.89391}+0.19267{\phi\:}_{p}^{0.32737}*{T}^{0.89391}$
Khodadadi et al.^23^	$\:\frac{{\mu\:}_{nf}}{{\mu\:}_{bf}}=11.938+0.258{e}^{{\phi\:}^{1.077}}-2.286{e}^{{T}^{0.266}}+0.679T$

## Materials and experimental equipment

### Graphene oxide (GO) nanofluid

In this study, Graphene oxide/water nanofluids were used, purchased at concentrations of 5–8 g/L. These nanofluids were purchased from Nano-meghyas Company, Mashhad, and their specifications are provided in Table 2. The graphene oxide/water nanofluid contains the surfactant Sodium Dodecyl Sulfate (SDS), which was added to prevent sedimentation and particle agglomeration. The samples were stored at room temperature, away from direct sunlight, and were gently stirred with a magnetic stirrer before measurements to ensure uniformity. All experiments were conducted within a few hours of opening the bottles to preserve the nanofluid’s physical properties. Viscosity was measured using a Brookfield DV-II+ viscometer, and each experiment was repeated at least three times. The precision of the instruments and the temperature control (± 0.1 °C) were considered in the uncertainty analysis to ensure reliable, accurate data.

**Table 2:** Characteristics of the purchased graphene oxide NF.

Chemical composition	CxHyOz
Concentration (g/l)	5,6,7,8
Morphology	Sheet
Thickness (nm)	Less than 2
Length (µm)	1–5
Color	Brown/black
Form	Liquid

**Fig. 1:**
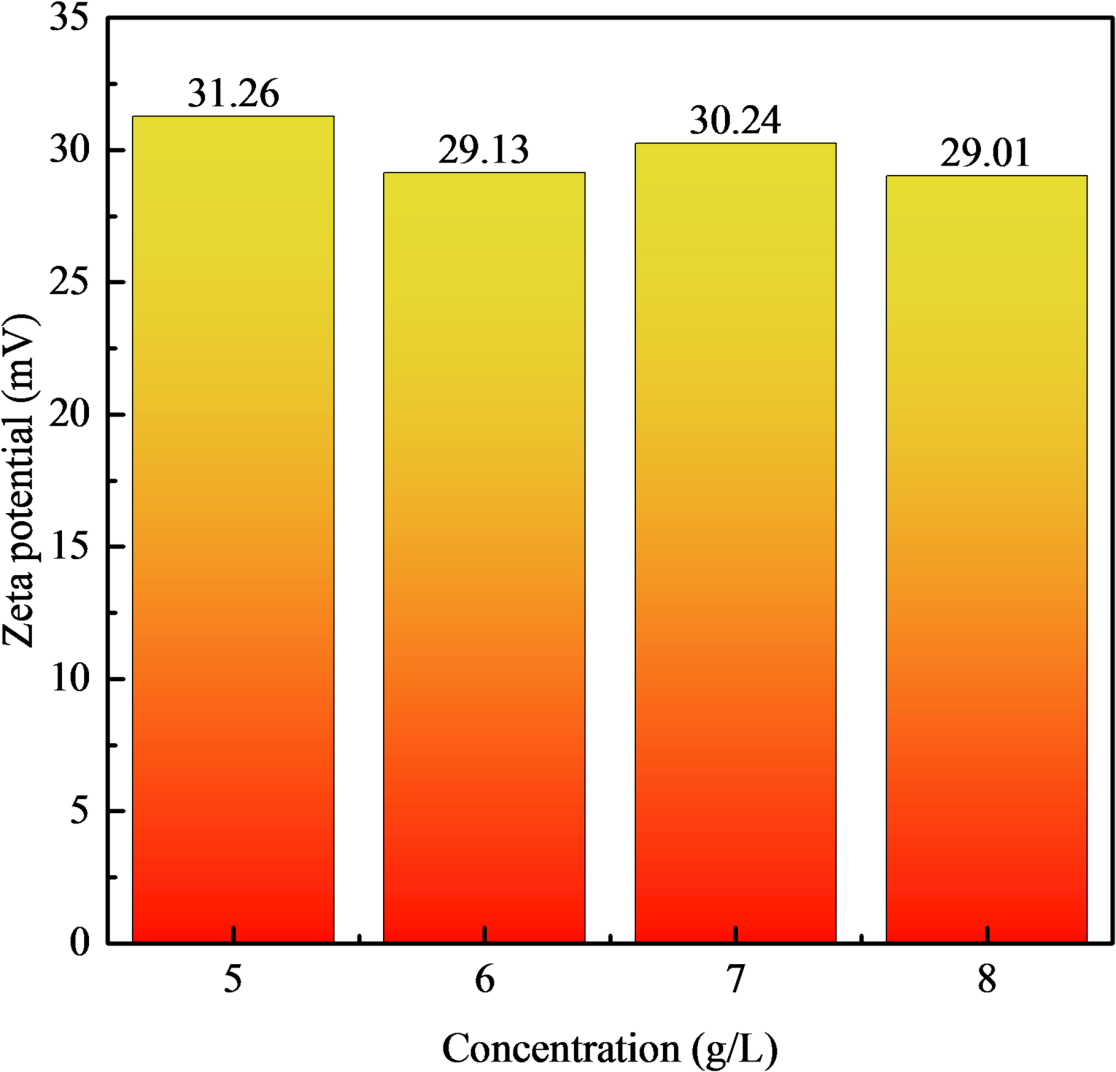
Absolute values of zeta potential of nanofluids at different concentrations and a fixed temperature of 25 °C.

**Fig. 2:**
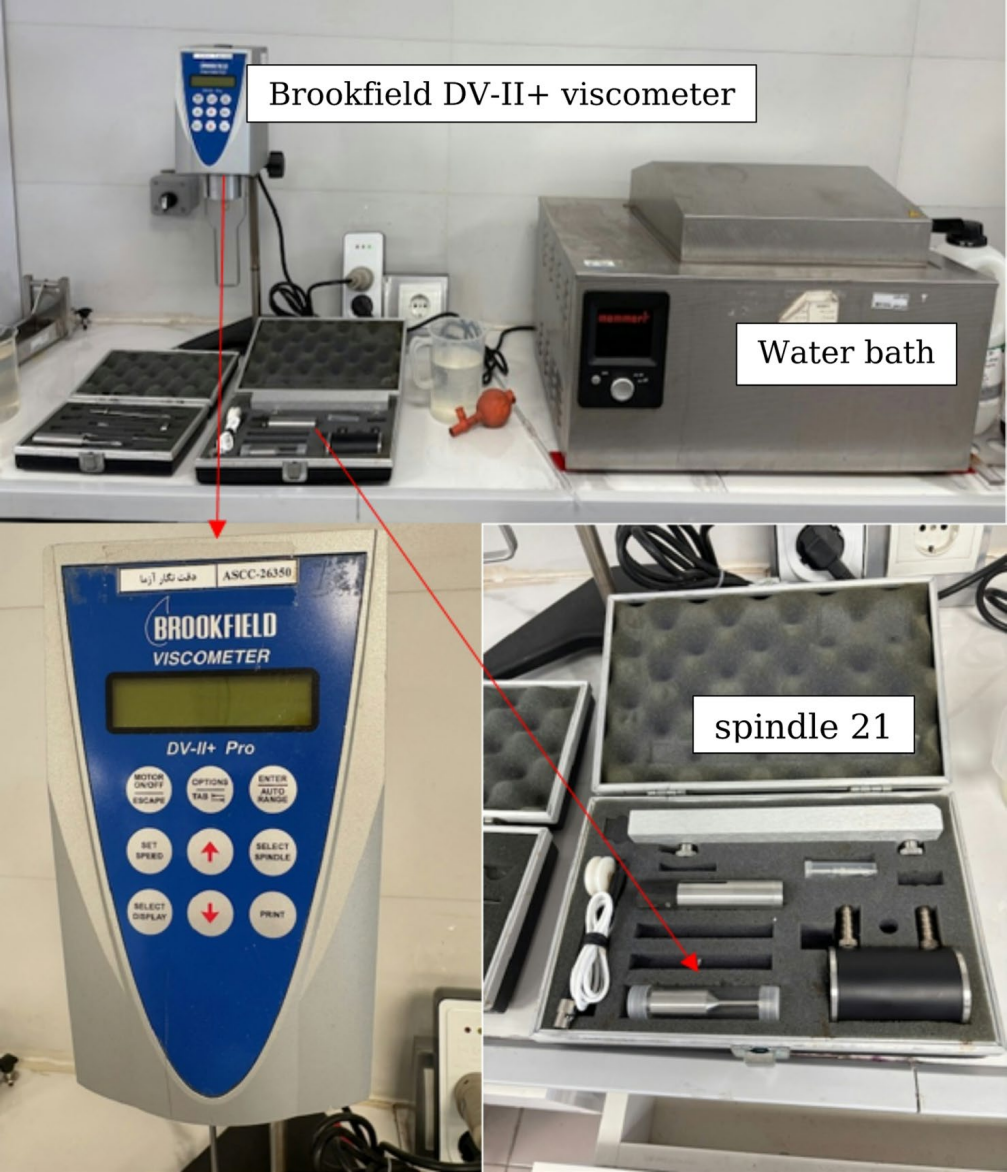
Experimental setup: Brookfield DV-II+ viscometer for nanofluid viscosity measurement.

The graphene oxide nanofluids used in this study were purchased ready-made from Nano Meghyas Company (Mashhad, Iran) and contained SDS surfactant to prevent sedimentation. Before measurements, the samples were stirred for 15 min using a magnetic stirrer at 500 rpm to ensure uniform distribution of nanoparticles in the base fluid. Although the manufacturer performed initial ultrasonication during synthesis to break agglomerates formed during storage and restore nanofluid stability, the samples were placed in an ultrasonic bath for 20 min at 100 W and 40 kHz. The temperature of the ultrasonic bath was controlled to 25–30 °C using an internal cooling system to prevent temperature rise and evaporation of the base fluid. The stability of the nanofluids after ultrasonication was confirmed by zeta potential measurements (Fig. 1), with values around 30 mV indicating good stability. All viscosity measurements were performed within 3 h of opening the original vials and completing the ultrasonication process to ensure the stability of the physical properties and prevent sedimentation. This is because we used standardized commercial nanofluids obtained from a reputable supplier, which does not disclose formulation details. Although this approach limits complete control over all variables, it offers an important advantage: the results of this study are directly generalizable and reliable for industrial users who typically utilize ready-made commercial nanofluids. To compensate for this limitation and ensure data reliability, we quantitatively and precisely monitored system stability, the primary function of the surfactant, throughout all experiments. Repeated zeta potential measurements (consistent values around 30 mV with variations less than ± 2 mV) and the high repeatability of viscosity results (standard deviation below 1.5%) provide strong evidence of uniform nanofluid stability during the testing period. These observations indicate that the surfactant’s effect on rheology was a consistent systematic influence, rather than an unknown random variable. Therefore, although the exact SDS concentration is not reported, its net effect is consistently observed across all data, and the proposed models (neural network and empirical correlation) accurately predict this stable behavior. Ensuring nanofluid stability was critical to the reliability of the viscosity modeling. Any sedimentation or nanoparticle agglomeration could have significantly altered the rheological properties, leading to inaccurate predictions by our models. Therefore, the rigorous preparation and stability monitoring processes described above were essential for validating the developed models. Furthermore, while we do not have the exact SDS surfactant concentration, the commercial and standardized nature of our nanofluid ensures its consistent presence across all samples. This means that any effect SDS has on viscosity acts as a constant factor across the data. Our models accurately capture and predict this stable, repeatable behavior, indicating that we have effectively controlled the parameters governing stability. The selection of the investigated concentration and temperature ranges in this study was based on scientific and experimental considerations related to the stability of graphene oxide nanofluids. From a scientific standpoint, at concentrations exceeding 8 g/L, van der Waals forces between graphene sheets increase, leading to particle agglomeration and physical instability of the nanofluid. This phenomenon not only causes rapid sedimentation but also leads to nonlinear behavior during viscosity measurements, thereby reducing the accuracy of the experimental data. Conversely, concentrations below 5 g/L provide an insufficient surface-to-volume ratio of nanoparticles, resulting in negligible changes in rheological and thermal properties relative to the base fluid. Therefore, the 5–8 g/L range was deliberately chosen to maintain nanofluid stability and homogeneity while ensuring measurable viscosity variations. Thermally, the temperature range of 20–35 °C was selected because previous studies^24,25^ reported that hydrogen bonds among the functional groups of graphene oxide weaken at temperatures above 40 °C, altering the network structure of the nanoparticles in Water. This transformation decreases stability, modifies rheological behavior, and can induce non-Newtonian characteristics, which would require a separate experimental design to analyze properly. In this study, a Brookfield DV-II+ viscometer was employed to measure the viscosity of the NF (Fig. 2). For more accurate viscosity measurements, the speed was set to 100 RPM, and 8 cc of the samples were placed in the sample holder using spindle 21 for viscosity analysis. The technical specifications of the viscometer used are listed in Table 3.

The sensitivity of the measurement instruments was carefully considered to ensure data accuracy. The viscometer used for viscosity measurements had a sensitivity of ± 0.001 Centipoise (cP), and its spindle was calibrated to ± 0.002 cP. The temperature was measured using a precision thermometer with an accuracy of ± 0.1 °C. These specifications were incorporated into the uncertainty analysis to evaluate their effect on the final viscosity data.

**Table 3:** Technical specifications of the viscometer used.

DV2EXTRA-Pro	Model
0.1-3000cP	Measurement range
10CC	Sample volume
1-200 RPM	| Speed range
1% of range	Accuracy
0.2%±	Repeatability
300 °C ≥ T≥-100 °C	Temperature range
9 kg	Net weight

To determine the device’s uncertainty, the measurement values were compared with reference values (Table 4). As shown, the uncertainty values are within ± 0.3. The viscometer measures the fluid’s resistance to the shear force generated by the spindle’s rotation.

**Table 4:** Measured values for device uncertainty.

Set point(RPM)	Reference (RPM)	Display (RPM)	Correction (RPM)	Uncertainty±(RPM)
5	5.1	5.0	+ 0.1	0.3
10	10.1	10.0	+ 0.1	0.3
20	20.1	20.0	+ 0.1	0.3
50	50.1	50.0	+ 0.1	0.3
100	100.2	100.0	+ 0.2	0.3

The total relative uncertainty in viscosity measurements was calculated using the root-sum-square (RSS) method, accounting for both temperature and viscometer measurement uncertainties. The repeatability uncertainty was estimated from repeated viscosity measurements under identical experimental conditions and was found to be very small compared with the instrumental uncertainties.

1. Temperature measurement accuracy: ±0.1 °C.
2. Viscometer accuracy: ±1.0%.
3. Relative uncertainty due to temperature: $\:\delta\:T/T=0.1/T$
4. Relative uncertainty due to the viscometer: $\:\delta\:\mu\:/\mu\:=0.01$
5. Repeatability uncertainty obtained from repeated measurements under identical conditions: U_rep_.
6. Total relative uncertainty: $\:\mathrm{U}{\upmu\:}$ = √(($\:\delta\:T/T$)^2^ + ($\:\delta\:\mu\:/\mu\:$)^2^)$\:+\:{\mathrm{U}}_{rep}$.

$$\:\delta\:T/T=0.1/20=0.005$$

$$\:{T=20^{\circ}{\mathrm{C}}\:\:\:\:\:\:\:\:\:\:\:\:\:\:\:\:\:\:\:\:\:\:\:\:\:\:\:\:\:\:\:\:\:\:\:\:\:U}_{\mu\:}=\sqrt{{0.01}^{2}+{0.005}^{2}}=\sqrt{0.000125}\approx\:0.01118\approx\:1.12\%$$

$$\:\delta\:T/T=0.1/25=0.004$$

$$\:T=25^{\circ}{\mathrm{C}}\:\:\:\:\:\:\:\:\:\:\:\:\:\:\:\:\:\:\:\:\:\:\:\:\:\:\:\:\:\:\:\:\:{U}_{\mu\:}=\sqrt{{0.01}^{2}+{0.004}^{2}}=\sqrt{0.000116}\approx\:0.01077\approx\:1.08\%$$

$$\:\delta\:T/T=0.1/30=0.00333$$

$$\:T=30^{\circ}{\mathrm{C}}\:\:\:\:\:\:\:\:\:\:\:\:\:\:\:\:\:\:\:\:\:\:\:\:\:\:\:{U}_{\mu\:}=\sqrt{{0.01}^{2}+{0.00333}^{2}}=\sqrt{0.0001101}\approx\:0.01049\approx\:1.05\%$$

$$\:\delta\:T/T=0.1/35=0.00286$$

$$\:T=35^{\circ}{\mathrm{C}}\:\:\:\:\:\:\:\:\:\:\:\:\:\:\:\:\:\:\:\:\:\:\:\:\:\:\:{U}_{\mu\:}=\sqrt{{0.01}^{2}+{0.00286}^{2}}=\sqrt{0.0001082}\approx\:0.0104\approx\:1.04\%$$

**Table 5:** Summary table of relative uncertainty.

Temperature (°C)	δT/T	Uµ (total uncertainty)
20	0.005	1.12%
25	0.004	1.08%
30	0.00333	1.05%
35	0.00286	1.04%

Table 5 shows that the total relative uncertainty remains low (≈ approximately 1%) across the examined temperature range (20–35 °C), ensuring that the viscosity measurements are reliable and precise. The slight decrease in uncertainty with temperature implies that high-temperature measurements are slightly more accurate relative to their true values, whereas at lower temperatures, temperature measurement error contributes more. These uncertainties are minor compared to the changes in viscosity caused by variations in nanoparticle concentration or temperature, and therefore do not affect the overall trends and conclusions of the study.

## Artificial neural network

**Fig. 3:**
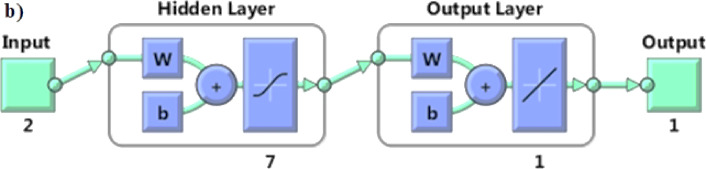
The architecture of the ANN used.

In this study, the properties of Graphene oxide NF were analyzed using an ANN model in MATLAB 2009. Temperature (°C) and Concentration (g/l) were used as inputs to the neural network, with the output being the NF viscosity to the base fluid (Fig. 3). The subsequent steps include determining the network’s number and type, training the network, selecting transfer functions, and defining the outputs. The evaluation and optimization of the ANN were carried out through trial and error. Various networks with different numbers of hidden layers were examined. The activation function used in the hidden layer was the Purlin function, and the Levenberg-Marquardt algorithm was used^26–28^.

In this study, an Artificial Neural Network (ANN) model was developed to predict the viscosity of a Graphene Oxide/water nanofluid as a function of temperature and nanoparticle concentration. The number of hidden layers and neurons was optimized through preliminary experiments by evaluating the Mean Squared Error (MSE) and the correlation coefficient (R^2^), and the 2-7-1 architecture was selected, meaning the network has two inputs (nanofluid temperature and nanoparticle concentration), one hidden layer with seven neurons, and one output (viscosity). Experimental data were divided into three sets: 70% for training, 15% for Validation, and 15% for testing. The validation set played a key role in preventing overfitting; the network was monitored and stopped when the validation error began to increase, ensuring it did not overfit the training data. Furthermore, to ensure model reliability and avoid reliance on random data splits, a 5-fold cross-validation was performed. In this method, the dataset was divided into five subsets, and the network was trained five times, each time using one subset as the test set and the remaining four as the training set. The results from each fold were combined to obtain final performance metrics, including MSE and R^2^, thereby ensuring the accuracy and stability of the predictions (Table 6). The tanh sigmoid transfer function was used in the hidden layer, and the purelin function was applied to the output neuron to accurately model the complex nonlinear relationships between inputs and outputs. The Levenberg-Marquardt (trainlm) algorithm was selected for network training due to its fast convergence and suitability for nonlinear regression problems with small to medium datasets. Network hyperparameters, including the number of hidden neurons, learning rate, and number of epochs, were optimized through repeated experiments and evaluation of MSE and R^2^ on the validation and test sets to achieve the best performance. The results demonstrated that the network accurately predicted nanofluid viscosity with minimal prediction error. This approach allowed the ANN model not only to validate experimental data but also to confirm the proposed empirical correlation, providing reliable predictions across different temperature and concentration conditions. Thus, the combination of experimental data, careful network design, hyperparameter optimization, cross-validation, and overfitting control offers a complete and reliable framework for modeling nanofluid viscosity, representing the main innovation of this study.

**Table 6:** 5-fold cross-validation results for the ANN model.

Fold	MSE	*R* ^2^
1	0.0021	0.9865
2	0.0023	0.9852
3	0.0022	0.9860
4	0.0020	0.9871
5	0.0022	0.9863
Average	0.00216	0.9862

The selection of the 2-7-1 topology for the artificial neural network was made based on a thorough optimization process. Several network structures with different numbers of hidden-layer neurons were evaluated to achieve the best balance between prediction accuracy, model complexity, and generalization capability. Seven neurons in the hidden layer provided the lowest MSE and MAE while exhibiting a very high correlation with the experimental data. In addition to accuracy, this topology effectively prevents overfitting, enabling the model to generalize well to new and out-of-sample data. Comparative tests revealed that increasing the number of neurons led to overfitting and reduced generalization, whereas decreasing the number of neurons reduced prediction accuracy. The 2-7-1 topology is also computationally efficient, reducing network training time. Therefore, this topology provides the best performance for modeling nanofluid viscosity and has strong scientific and practical justification.

## Results and evaluation

### XRD, FESEM, and zeta potential images

**Fig. 4:**
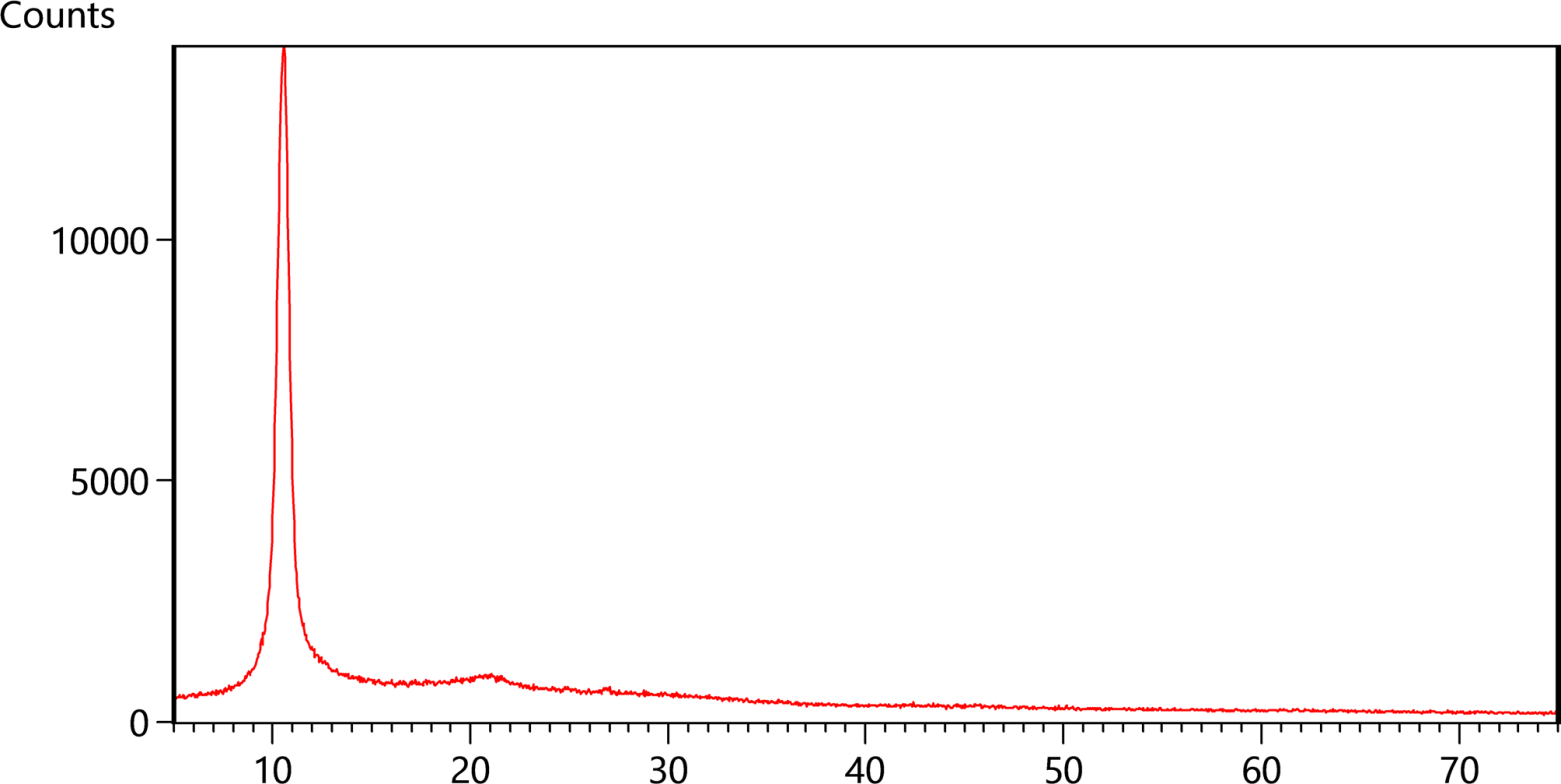
XRD image of nanoparticles.

**Fig. 5:**
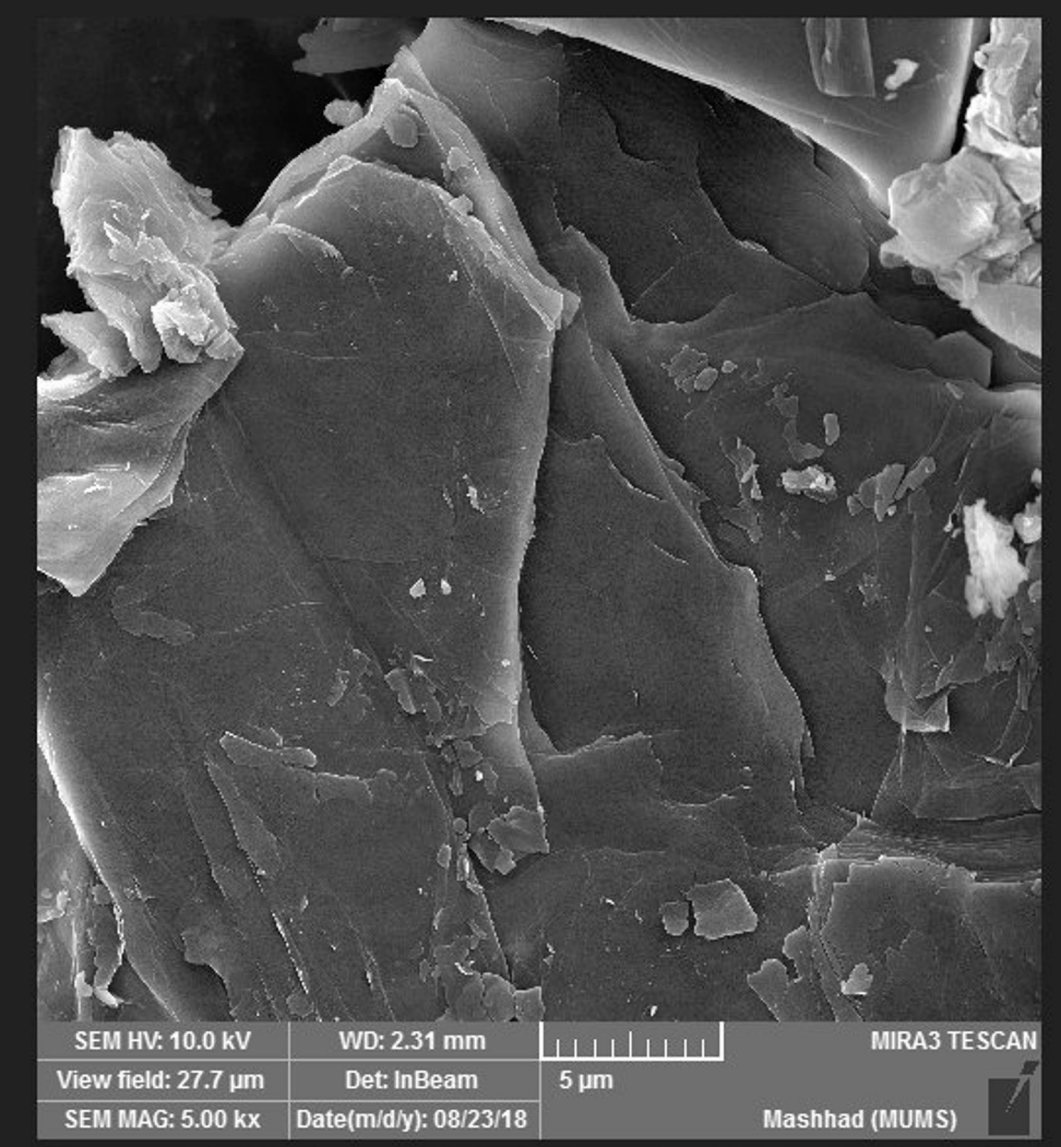
FESEM image of nanoparticles.

The Graphene oxide nanoparticles prepared at various concentrations were analyzed for their surface and atomic structure using X-ray Diffraction (XRD) and Field Emission Scanning Electron Microscopy (FESEM) images, as shown in Figs. 4 and 5. The results indicated that the nanoparticles’ atomic characteristics were in good agreement with previous studies^29^, with an average nanoparticle size of less than 2 nm. Additionally, Fig. 5 illustrates that the nanoparticles have a layered structure.

To evaluate the zeta potential, a CAD model device from France was used. According to American Society for Testing and Materials (ASTM) standards, a zeta potential range of 20 to 30 millivolts indicates moderate stability, while values greater than ± 30 millivolts are considered to represent high stability. The zeta potential of the nanofluid at a concentration of 6 g/L was measured at 29.13 mV in this study, indicating good stability of the nanoparticles in the aqueous solution. The zeta potential value is a key indicator of NF stability. According to Fig. 1, the absolute value of the zeta potential for all samples studied in this work is approximately 30 mV, indicating sufficient electrostatic stability of the nanofluid suspensions within the experimental conditions. The sample with the highest concentration (8 g/L) shows a slightly lower zeta potential magnitude. This reduction can be attributed to increased particle-particle interactions and the higher risk of nanoparticle agglomeration at relatively high nanofluid concentrations.

In fact, zeta potential monitoring across all tested temperature and concentration conditions was performed as part of the quality control protocol in the present study; however, due to manuscript length limitations, only representative data at a concentration of 6 g/L were reported. Given the importance of this point, the previously measured temperature-dependent stability data are presented in Table 7, with the corresponding analysis provided below. These data and the related analysis will certainly be incorporated into the revised version of the manuscript.

**Table 7:** Zeta potential values (mV) of graphene oxide nanofluid under different conditions.

Concentration (g/L)	20 °C	25 °C	30 °C	35 °C
5	− 29.4 ± 0.8	− 28.9 ± 0.7	− 28.1 ± 0.9	− 27.5 ± 1.0
6	− 29.8 ± 0.6	− 29.13 ± 0.7	− 28.7 ± 0.8	− 28.0 ± 0.9
7	− 30.5 ± 0.5	− 30.0 ± 0.6	− 29.2 ± 0.7	− 28.6 ± 0.8
8	− 31.2 ± 0.7	− 30.6 ± 0.8	− 29.9 ± 0.9	− 29.2 ± 1.1

Analysis of the data in the table reveals that all zeta potential values, even at the highest tested temperature (35 °C), remain within the highly stable range (absolute value greater than 25 mV). This clearly confirms that the nanofluid exhibited no significant instability or aggregation throughout all viscosity experiments. Furthermore, a systematic, temperature-dependent trend is observed: as temperature increases, the absolute zeta potential decreases mildly and predictably. This behavior is consistent with the thermodynamic compression of the electrical double layer due to increased thermal energy. Importantly, this decrease never brings the system close to the instability threshold. On the other hand, at each constant temperature, increasing nanoparticle concentration leads to a higher charged surface intensity, thereby increasing the absolute zeta potential, indicating better electrostatic stability at higher concentrations. In conclusion, these supplementary data clearly demonstrate that nanofluid stability was a controlled and consistent parameter throughout the study. Therefore, the measured viscosity variations can be confidently attributed to the direct effects of temperature and nanoparticle concentration, rather than to factors arising from instability.

### Validation

**Fig. 6:**
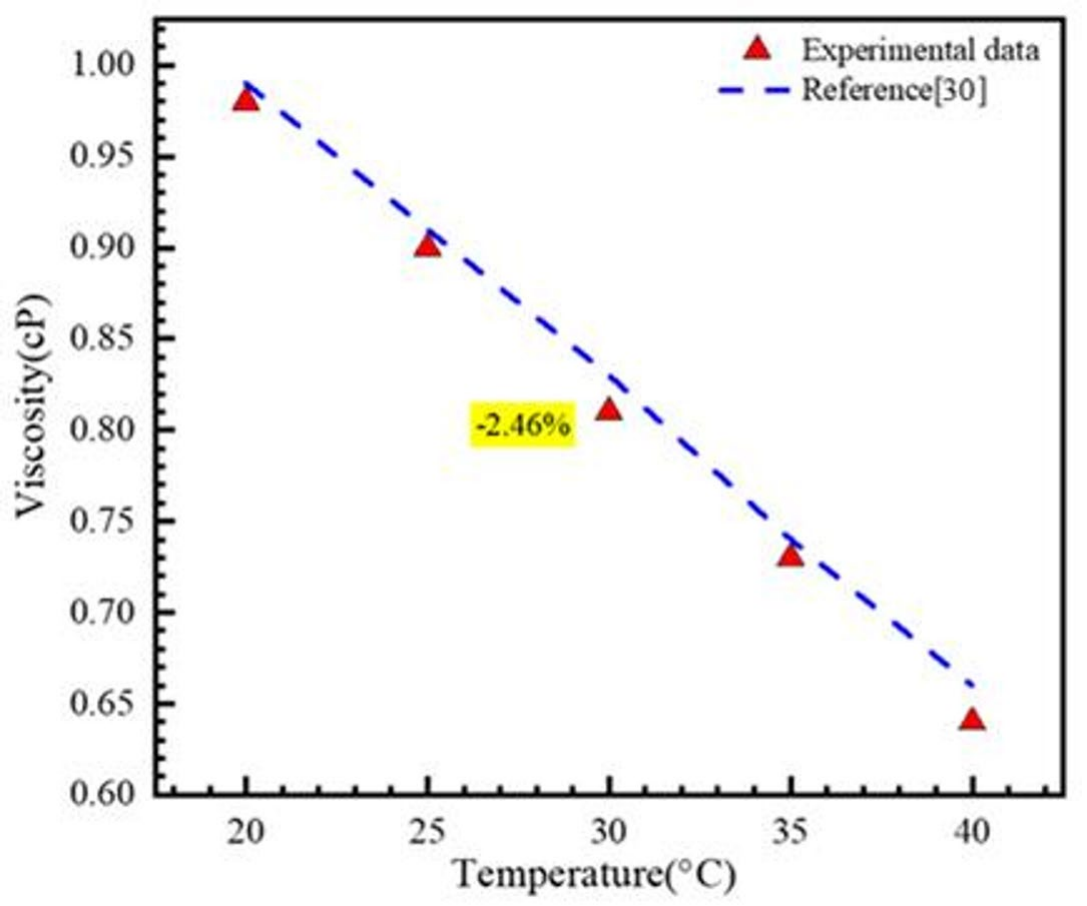
Validation of experimental data for water-based fluid with reference values^30^.

To ensure the accuracy of the Brookfield viscometer, the viscosity of the base fluid was measured and compared with the standard viscosity of Water^30^ (Fig. 6). The results indicate that the viscosity measurement error falls within the device’s acceptable range, ensuring the reliability of the measurement method and the device’s accuracy. The maximum error observed during this Validation was 2.46% at 30 °C, reflecting the accuracy of the viscometer’s data. Additionally, the dynamic viscosity of Water decreased with increasing temperature.

**Fig. 7:**
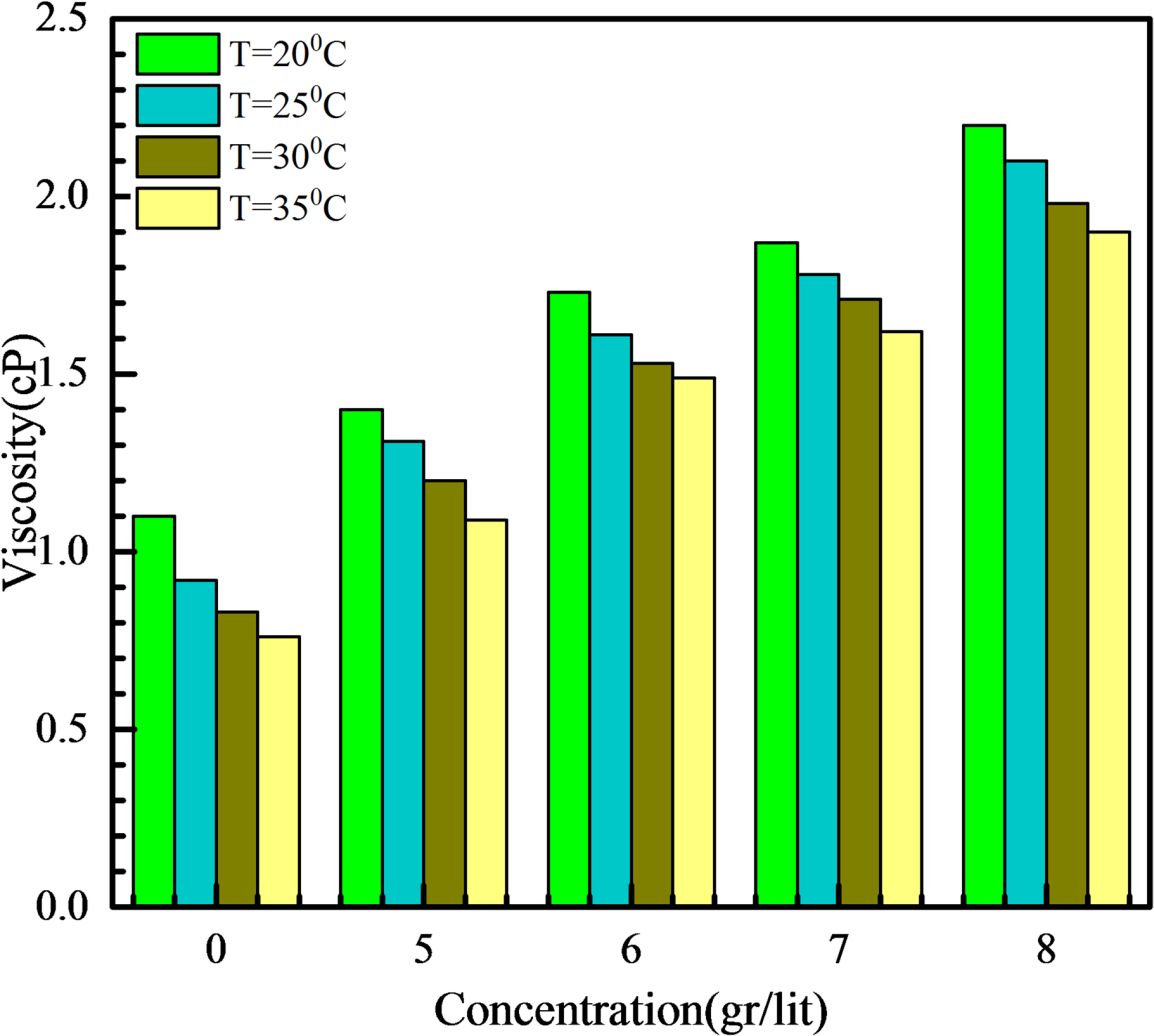
Experimental viscosity values at various temperatures for graphene oxide NF.

**Fig. 8:**
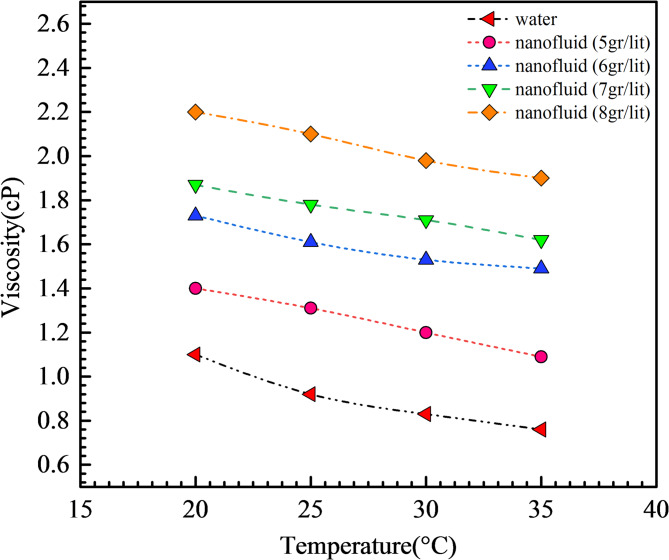
Experimental variation of viscosity with temperature for graphene oxide NF.

**Fig. 9:**
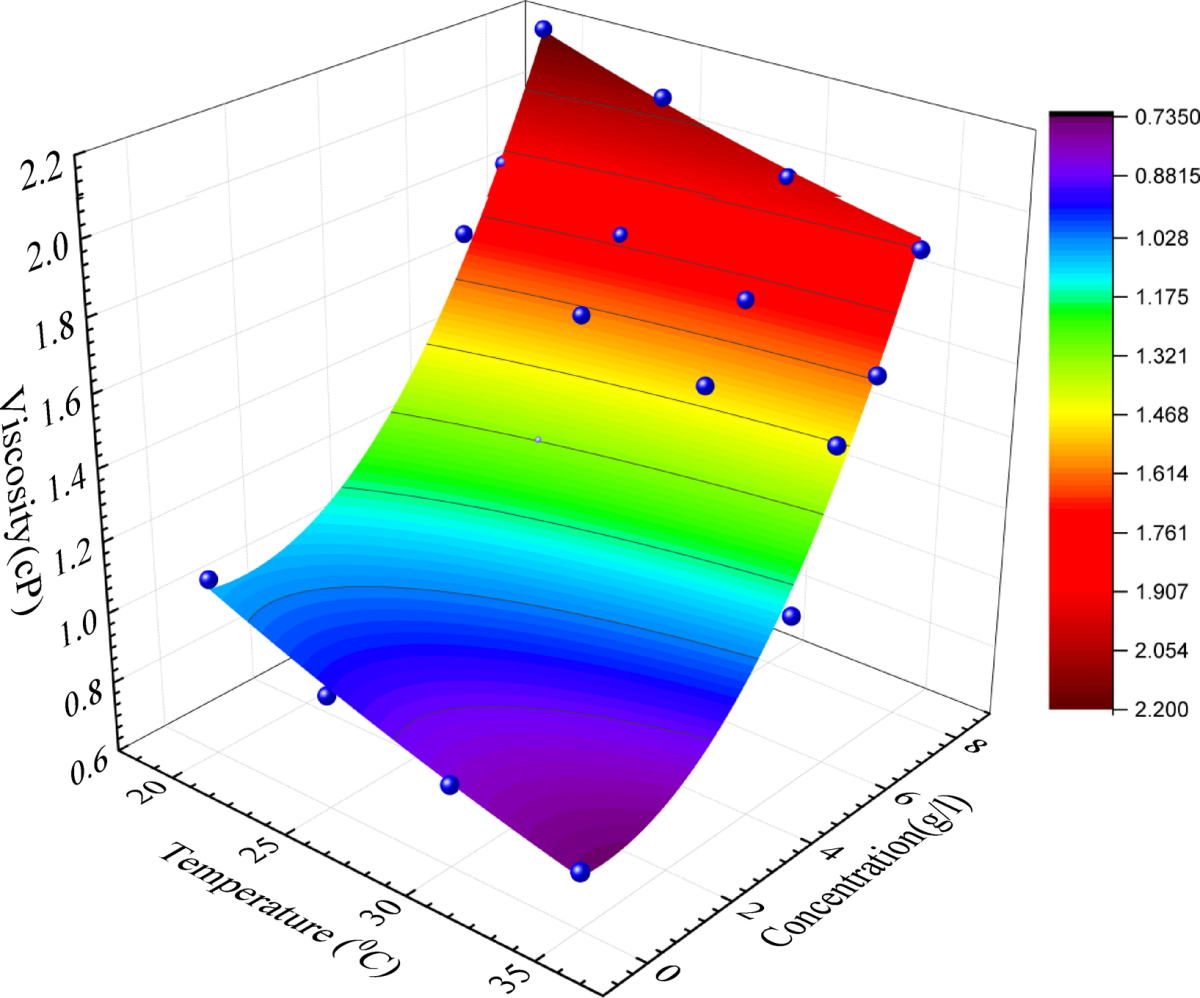
Three-dimensional comparison between experimental data based on temperature and concentration, and the fitted surface for obtaining the proposed relationship.

Figures 7 and 8 illustrate the viscosity variation of Graphene oxide nanofluids at different concentrations and temperatures. As shown in Fig. 7, the slope of viscosity change with concentration is significantly steeper at lower temperatures compared to higher temperatures, indicating a decreased sensitivity of viscosity to concentration changes at elevated temperatures. For instance, at 20 °C, the viscosity of the water-based fluid is 1.1 cP, whereas the NF with a concentration of 5 g/L has a viscosity of 1.4 cP. Temperature and concentration directly influence the viscosity of nanofluids. As temperature increases, van der Waals forces between molecules weaken, making intermolecular bonds easier to break and reducing viscosity. Figure 9 depicts the viscosity of graphene oxide nanofluids as a function of the two main parameters shown in the figure. Increasing the concentration raises the viscosity of the Graphene NF. The addition of particles increases the friction and resistance of the fluid layers to motion, thereby increasing the fluid’s internal shear stress. Consequently, an increase in viscosity is expected.

To assess the rheological behavior of the samples, viscosity measurements were performed using different spindles and over a range of rotational speeds. The obtained results indicated that viscosity variations across the applied shear-rate range followed a linear trend, with no significant dependence on shear rate. This observation confirms that the samples exhibited approximately Newtonian behavior under the experimental conditions. These findings are also consistent with results reported in the literature. For example, Kamatchi et al.^31^ have shown that similar samples at various temperatures and concentrations exhibit fully Newtonian behavior, with viscosity varying linearly with shear stress. Based on this agreement, the use of a constant spindle speed in the measurements is scientifically justified.

## Estimation relationship

### Proposed estimation relationship for graphene oxide NF viscosity

Theoretical models from past studies, such as those by Einstein and Brinkman, have sometimes shown limitations in predicting NF properties. In this research, a new relationship is proposed to enhance accuracy in predicting the viscosity of Graphene oxide/water NF across various concentrations and temperatures. This relationship was derived by curve fitting the experimental data in Origin 2017, yielding an R^2^ of 0.98704 and an MSE of 0.002188, indicating the high accuracy of the proposed model (Fig. 9). The proposed relationship is shown in Eq. 1. This equation includes constant coefficients and is a function of temperature, concentration, temperature squared, concentration squared, and the interaction of temperature and concentration (Table 8). The standard errors and correlation coefficients for each coefficient are provided; coefficients a and c have correlation coefficients of 0.99975 and standard errors of 0.00005, respectively, which are satisfactory. The coefficients with relatively high p-values were examined. Since the $\:{\mathrm{T}}^{2}$ and $\:\mathrm{T}{\upphi\:}\:$ terms did not contribute significantly to describing the observed trend in the experimental data, these terms were removed to reduce unnecessary model complexity and avoid the inclusion of statistically weak parameters. Consequently, the revised empirical model includes only the linear effect of temperature and the linear and quadratic effects of nanoparticle concentration, along with a constant term. This simplified form still captures the overall trend of viscosity variation within the investigated experimental range, while also providing a clearer physical interpretation of the remaining coefficients. Furthermore, the revised manuscript emphasizes that the proposed correlation is empirical and valid within the experimental temperature (20–35 °C) and nanoparticle concentration (5–8 g/L) ranges; therefore, extrapolation beyond these ranges should be applied with caution.

1 $$\:{{\upmu\:}}_{\mathrm{n}\mathrm{f}}={\mathrm{z}}_{0}+\mathrm{a}\mathrm{T}+\mathrm{b}{\upphi\:}+\mathrm{d}{{\upphi\:}}^{2}\:$$

**Table 8:** Coefficients of the proposed estimation relationship.

	Value	Estimation Error	t-Value	Prob>|t|	*R* ^2^
z0	1.77616	0.38627	4.59826	4.14E-04	0.99895
a	-0.04276	0.02791	-1.53211	0.14778	0.99975
b	-0.03899	0.02749	-1.41823	0.178	0.99406
c	3.80E-04	5.00E-04	0.75984	0.45995	0.99913
d	0.02114	0.0021	10.06635	8.61E-08	0.97894
f	5.20E-04	8.03E-04	0.64717	0.52799	0.99115
R^2^ (COD)	0.98704				
Adj. R^2^	0.98241				

To check how accurately the proposed model and ANN are consistent with the experimental results, the margin of deviation is used, as given by the following equation^32^:

2 $$\:\mathrm{M}\mathrm{a}\mathrm{r}\mathrm{g}\mathrm{i}\mathrm{n}\:\mathrm{o}\mathrm{f}\:\mathrm{D}\mathrm{e}\mathrm{v}\mathrm{i}\mathrm{a}\mathrm{t}\mathrm{i}\mathrm{o}\mathrm{n}\left(\mathrm{M}\mathrm{O}\mathrm{D}\right)\:\left({\%}\right)=\frac{{\mu\:}_{nf\left(\mathrm{C}\mathrm{o}\mathrm{r}\mathrm{r}\:\mathrm{o}\mathrm{r}\:\mathrm{A}\mathrm{N}\mathrm{N}\right)}-{\mu\:}_{Exp}}{{\mu\:}_{Exp}}*100$$

1. Physical interpretation of coefficients:

The proposed empirical correlation (Eq. 1) is expressed as a polynomial function of temperature (T) and nanoparticle concentration (φ). In the revised model, only statistically significant terms were retained to obtain a simpler, more interpretable relationship among viscosity, temperature, and concentration. The physical interpretation of the remaining coefficients is summarized as follows:

- **Coefficient a (linear temperature coefficient)**:

This coefficient represents the direct effect of temperature on viscosity. Its negative value indicates that viscosity decreases with increasing temperature. This behavior is consistent with the well-known principles of fluid dynamics, in which increasing temperature enhances molecular mobility and reduces intermolecular resistance to flow.

- **Coefficient b (linear concentration coefficient)**:

This coefficient reflects the influence of nanoparticle concentration on the nanofluid’s viscosity. The addition of nanoparticles introduces extra resistance to fluid motion due to particle–fluid and particle–particle interactions.

- **Coefficient d (quadratic concentration coefficient)**:

The positive value of this coefficient indicates that viscosity increases nonlinearly with concentration. This behavior is commonly observed in nanofluids and can be attributed to stronger particle interactions and microstructural effects at higher nanoparticle concentrations.

2. Applicability and extrapolation:

It should be noted that the proposed correlation is an empirical model derived from experimental data and is intended to describe the viscosity behavior within the investigated range of conditions (temperature 20–35 °C and nanoparticle concentration 5–8 g/L). Therefore, the application of this correlation outside the experimental range should be treated with caution. This clarification has been included in the revised manuscript to avoid any misunderstanding regarding the applicability of the proposed model.

3. Limitations.

It is emphasized that the correlation is based on the available experimental dataset. For wider temperature or concentration ranges, additional experiments and further model development would be required to ensure reliable prediction accuracy.

**Fig. 10:**
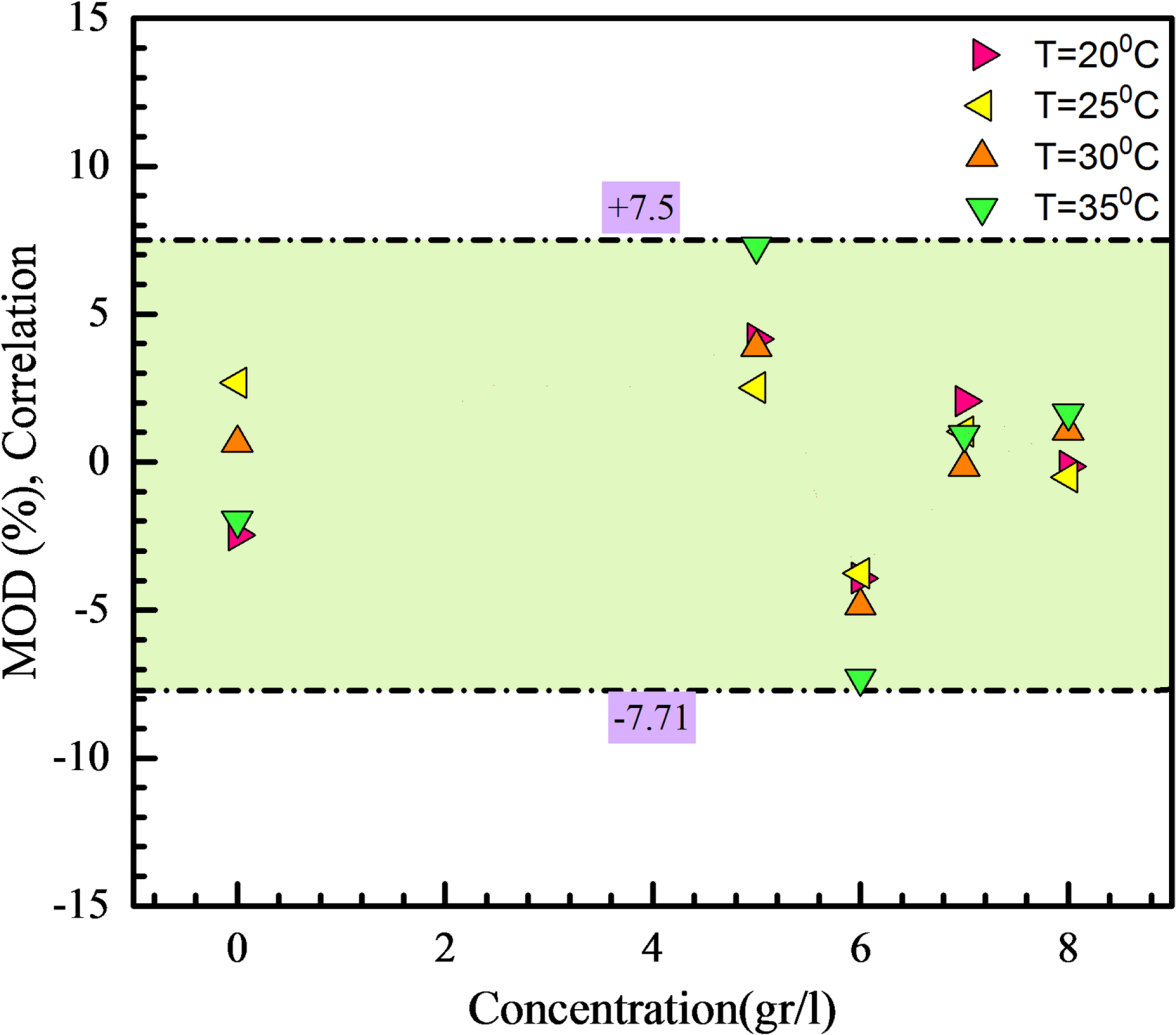
MOD for experimental data with the proposed relationship.

**Fig. 11:**
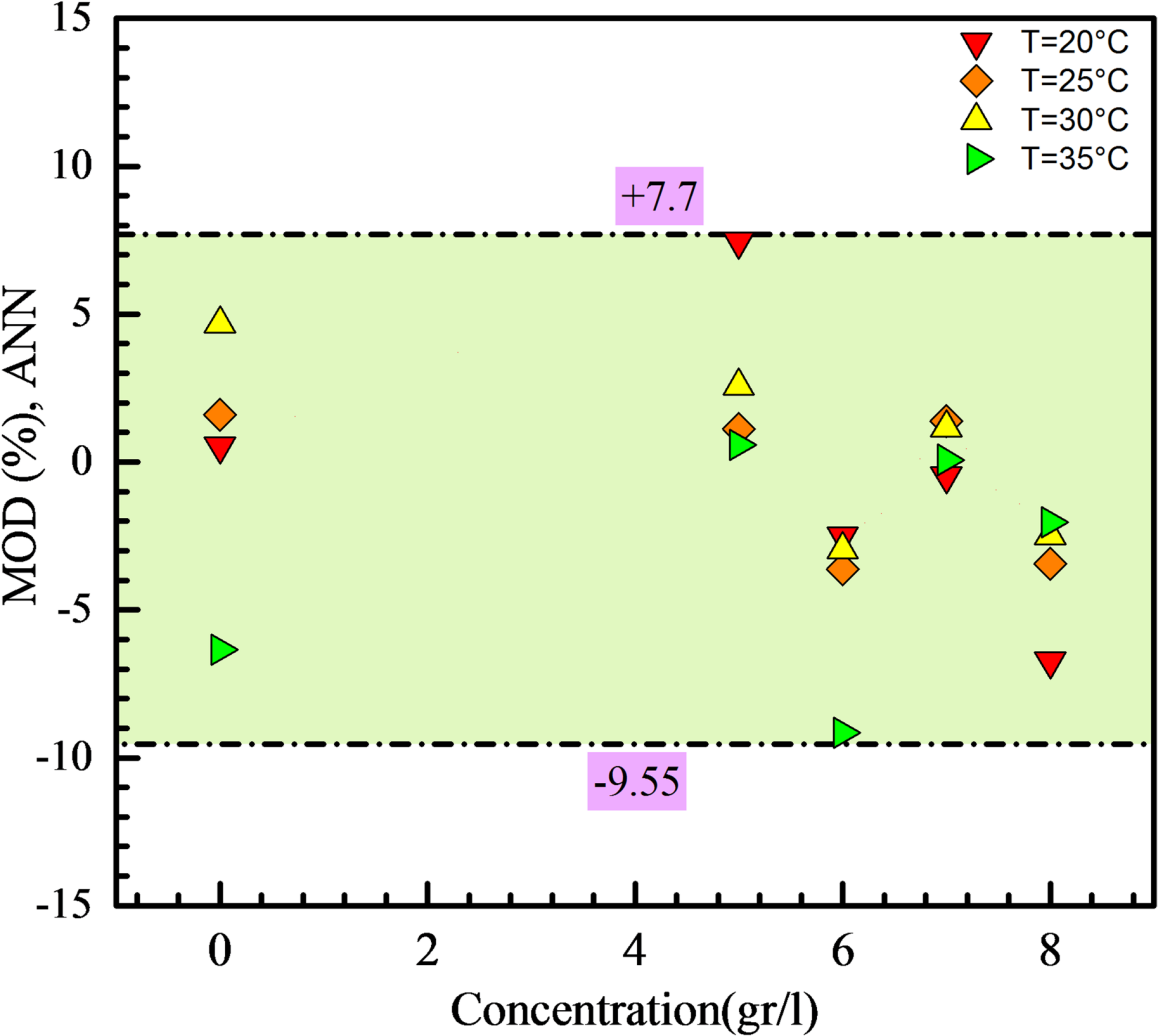
The MOD for experimental data with the proposed ANN.

Figures 10 and 11 illustrate the MOD as a function of concentration and temperature for both the proposed model and the ANN. As shown in Fig. 10, the proposed model exhibits a maximum MOD of + 7.8% at 35 °C with a NF concentration of 5 g/L, and a minimum MOD of -7.71% at 35 °C with a concentration of 6 g/L. Figure 11 indicates that for the ANN data, the maximum MOD is 8% at 30 °C with a concentration of 5 g/L, and the minimum MOD is -9.55% at 35 °C with a concentration of 6 g/L. In summary, smaller deviations from the zero line indicate higher accuracy, reflecting the performance of both the proposed model and the neural network.

**Fig. 12:**
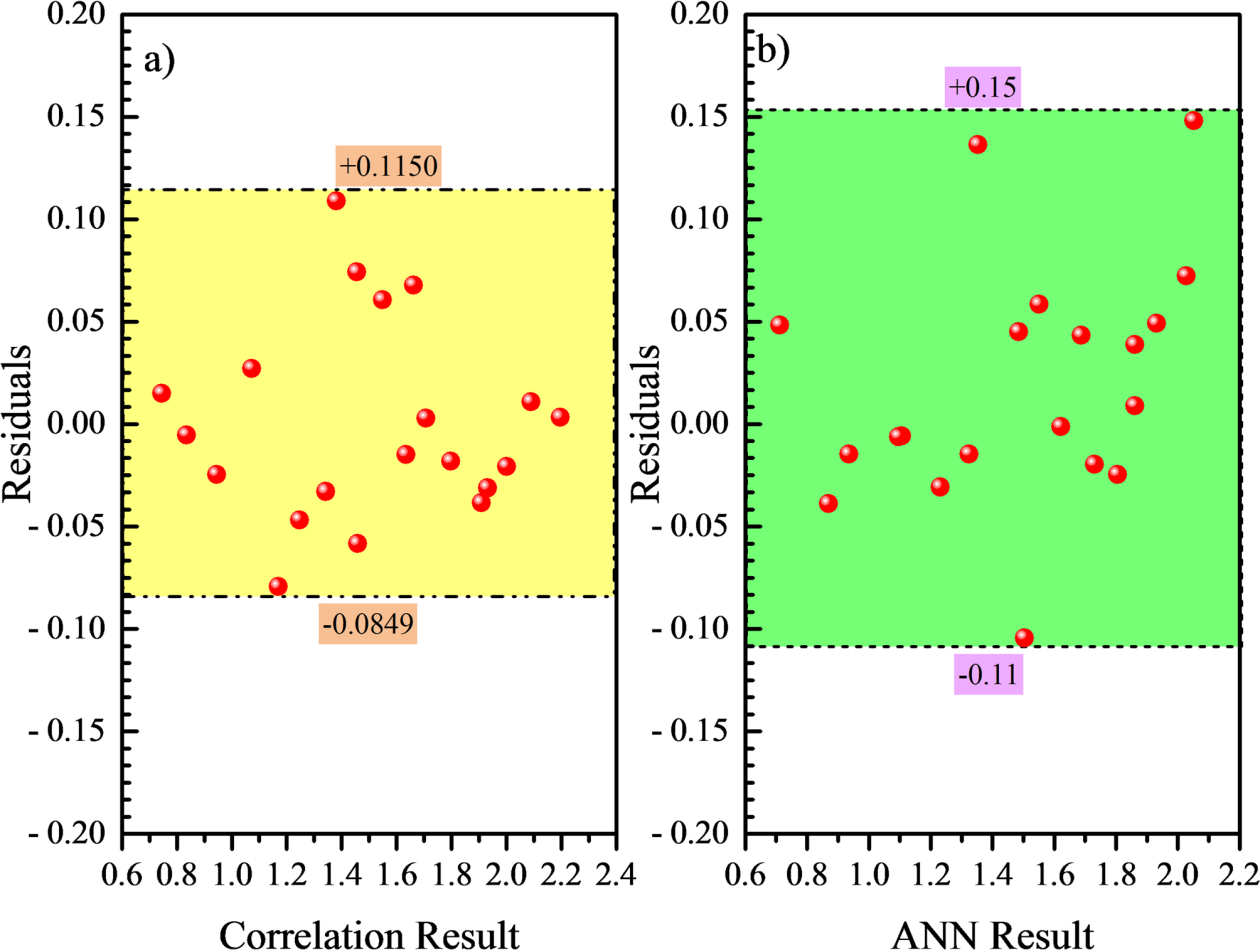
Residual for experimental data with the (**a**) proposed relationship and (**b**) ANN.

Figure 12a, b shows the residuals for the experimental data compared with those predicted by the proposed relationship and the ANN. The residual values represent the discrepancies between experimental viscosity measurements and the predictions made by the proposed model and the ANN. For the proposed relationship, the maximum and minimum residual values are + 0.1150 and − 0.0849, respectively. For the ANN, these values are 0.15 and − 0.11. Residuals closer to zero indicate higher accuracy of the proposed model and the ANN in predicting the experimental data.

To examine the normality of the residual distribution, two well-established statistical tests, namely the Shapiro-Wilk and Kolmogorov-Smirnov tests, were employed. The Shapiro-Wilk test yielded p-values of 0.214 and 0.326 for the empirical model and the ANN residuals, respectively, both exceeding the 0.05 significance level. The Kolmogorov-Smirnov test also confirmed the normality of residual distribution (p-value > 0.05). Therefore, the null hypothesis that the residuals are normally distributed cannot be rejected, confirming that the residuals from both models are normally distributed.To investigate the absence of systematic bias in predictions, a one-sample t-test was conducted. The mean residual values were − 0.0012 for the empirical model and − 0.0008 for the ANN model. The t-test results indicated that these values do not differ significantly from zero (p-value > 0.05), confirming that both models are free from systematic bias. Additionally, the Durbin-Watson test was performed to examine serial correlation among residuals, yielding a statistic of 1.98. This value is very close to 2, indicating no autocorrelation in the residuals. As observed in Fig. 12a,b of the manuscript, the residuals are randomly scattered around the zero line, with no discernible pattern, such as an increase in variance with increasing predicted values. This visual observation is now quantitatively confirmed by the statistical tests mentioned above. To further complement the analysis, Q-Q plots of the residuals were also generated, showing that the points align well with the normal line, providing additional visual confirmation of normality. In conclusion, the results of the statistical tests clearly demonstrate that the residuals of both models (empirical correlation and ANN) follow a normal distribution, are free from systematic bias, and exhibit no autocorrelation. These findings validate the robustness of the proposed models and the accuracy of their predictions.

To evaluate the Mean squared error(MSE), Mean Absolute Error(MAE), Root-mean-square deviation (RMSE), and Coefficient of determination (R^2^), relations 3 to 6 were used^33–35^.

3 $$\:\mathrm{M}\mathrm{S}\mathrm{E}=\frac{1}{\mathrm{N}}\sum\:_{\mathrm{i}=1}^{\mathrm{n}}{({\mathrm{Y}}_{\mathrm{i}\mathrm{j}}-{\mathrm{X}}_{\mathrm{i}\mathrm{j}})}^{2}$$

4 $$\:\mathrm{M}\mathrm{A}\mathrm{E}=\frac{1}{\mathrm{N}}\sum\:_{\mathrm{i}=1}^{\mathrm{n}}({\mathrm{Y}}_{\mathrm{i}\mathrm{j}}-{\mathrm{X}}_{\mathrm{i}\mathrm{j}})$$

5 $$\:\mathrm{R}\mathrm{M}\mathrm{S}\mathrm{E}=\sqrt{\frac{1}{\mathrm{N}}\sum\:_{\mathrm{i}=1}^{\mathrm{n}}{({\mathrm{Y}}_{\mathrm{i}\mathrm{j}}-{\mathrm{X}}_{\mathrm{i}\mathrm{j}})}^{2}}$$

6 $$\:{R}^{2}=\frac{\sum\:_{\mathrm{i}=1}^{\mathrm{n}}{\left({\mathrm{Y}}_{\mathrm{i}\mathrm{j}}-\stackrel{-}{\mathrm{Y}}\right)}^{2}\:-\sum\:_{i=1}^{n}{\left({\mathrm{X}}_{\mathrm{i}\mathrm{j}}-\stackrel{-}{\mathrm{X}}\right)}^{2}\:\:\:}{\sum\:_{i=1}^{n}{\left({\mathrm{Y}}_{\mathrm{i}\mathrm{j}}-\stackrel{-}{\mathrm{Y}}\right)}^{2}}$$

where $\:{\mathrm{Y}}_{\mathrm{i}\mathrm{j}}$ is the ith experimental data, $\:{\mathrm{X}}_{\mathrm{i}\mathrm{j}}$ is the predicted value, and n is the number of observations.

**Fig. 13:**
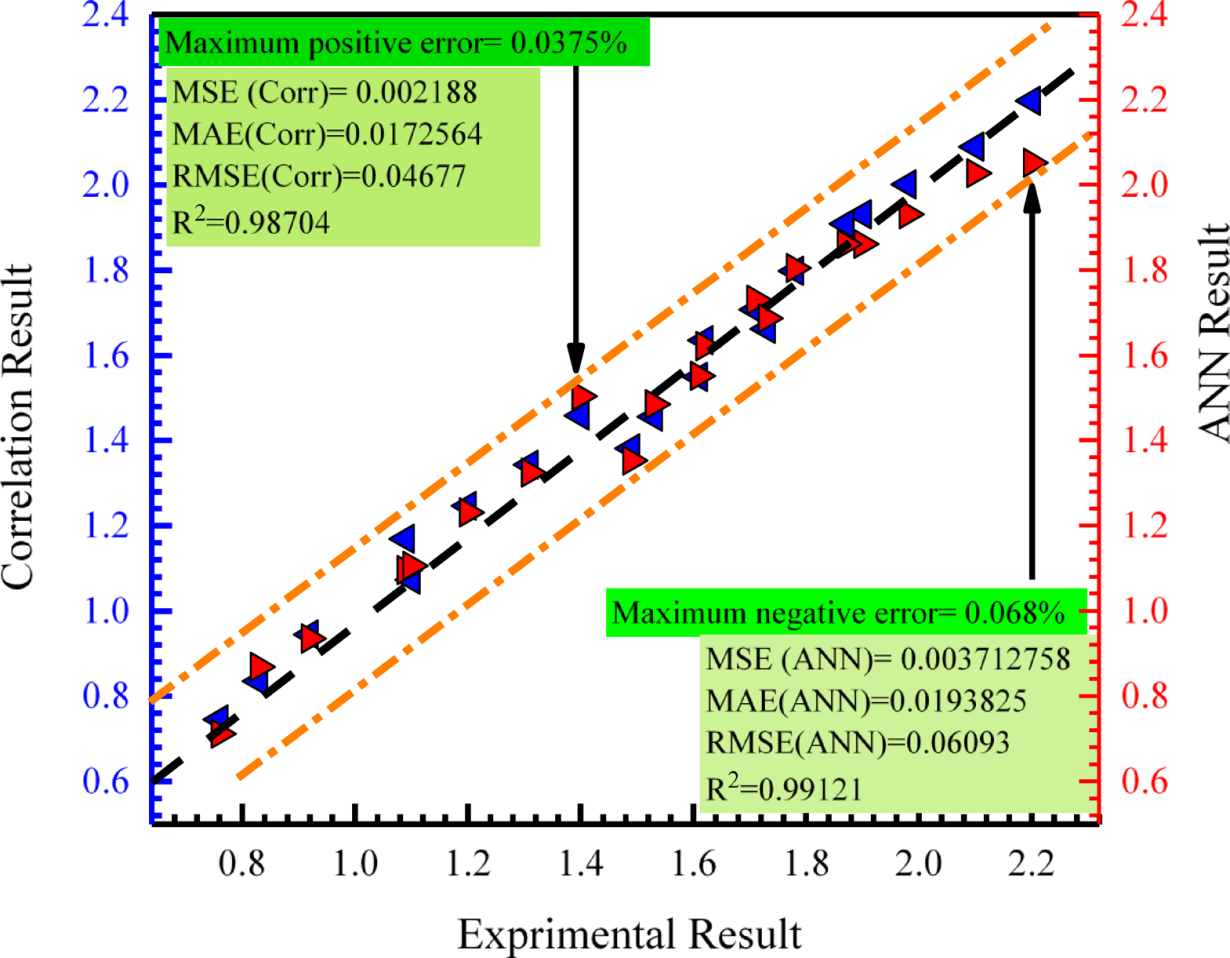
A comparison between the experimental findings and the predicted outcomes.

**Fig. 14:**
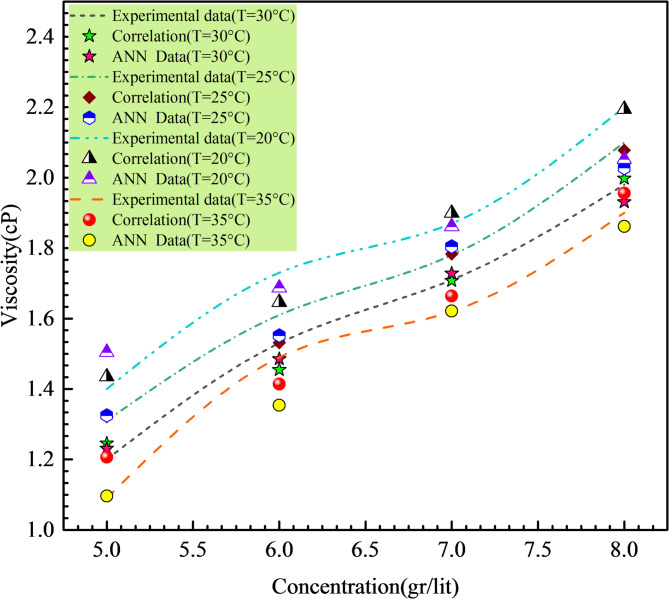
Predicted data by the neural network and proposed relationship compared with experimental data for graphene oxide NF at different temperatures.

A precise comparison between experimental results, the proposed relationship (Eq. 1), and the Perceptron ANN for Graphene oxide NF viscosity is shown in Fig. 13. Most data points for both the proposed relationship and the ANN are situated on or near the 45-degree line, indicating high accuracy. The MSE, MAE, RMSE, and R^2^ for the proposed relationship and the ANN are 0.002188, 0.0172564, 0.04677, 0.98704, and 0.003712758, 0.0193825, 0.06093, and 0.99122, respectively, reflecting successful evaluation. The maximum positive and negative errors for the data are 0.0375% and 0.068%, respectively. Comparisons between the experimental data, the proposed relationship, and the ANN results are presented in Fig. 14 for temperatures ranging from 20 °C to 35 °C and concentrations from 0 g/L to 8 g/L. This figure demonstrates that the experimental data align well with both the proposed relationship and the ANN, with minor discrepancies observed at 35 °C for concentrations of 5 and 6 g/L. These small deviations are attributed to experimental errors and human error.

To ensure reliable predictive performance and prevent potential overfitting arising from the limited dataset, the ANN model was carefully designed and evaluated through multiple complementary strategies. The 2-7-1 architecture was selected after extensive trial-and-error assessments, in which increasing the number of hidden neurons led to overfitting (characterized by rising validation error), while reducing it caused noticeable loss of predictive accuracy. An early-stopping criterion was implemented by allocating 15% of the data for validation, allowing training to terminate as soon as the validation error began to increase. In addition, a 5-fold cross-validation procedure was conducted, yielding highly consistent performance metrics (MSE = 0.0022 and R^2^ = 0.987), confirming the model’s stability and generalization capability. Furthermore, the available experimental dataset provides a sufficient number of observations relative to the network’s learnable parameters, helping mitigate the risk of overfitting. Moreover, strong agreement between the ANN predictions and the independently developed empirical correlation further verifies that no overfitting behavior is present.

**Fig. 15:**
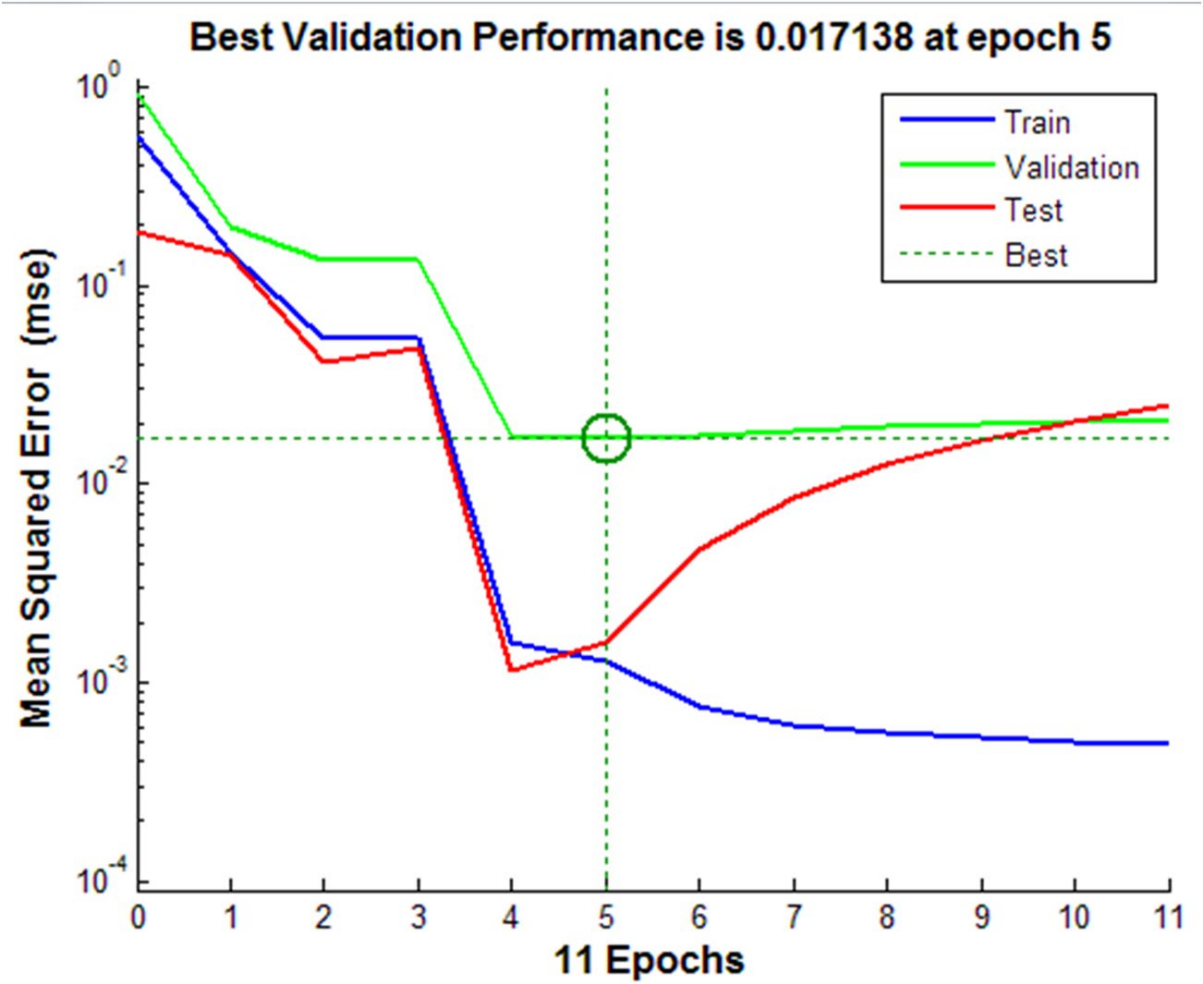
The MSE for all data sets using the Purelin function.

**Fig. 16:**
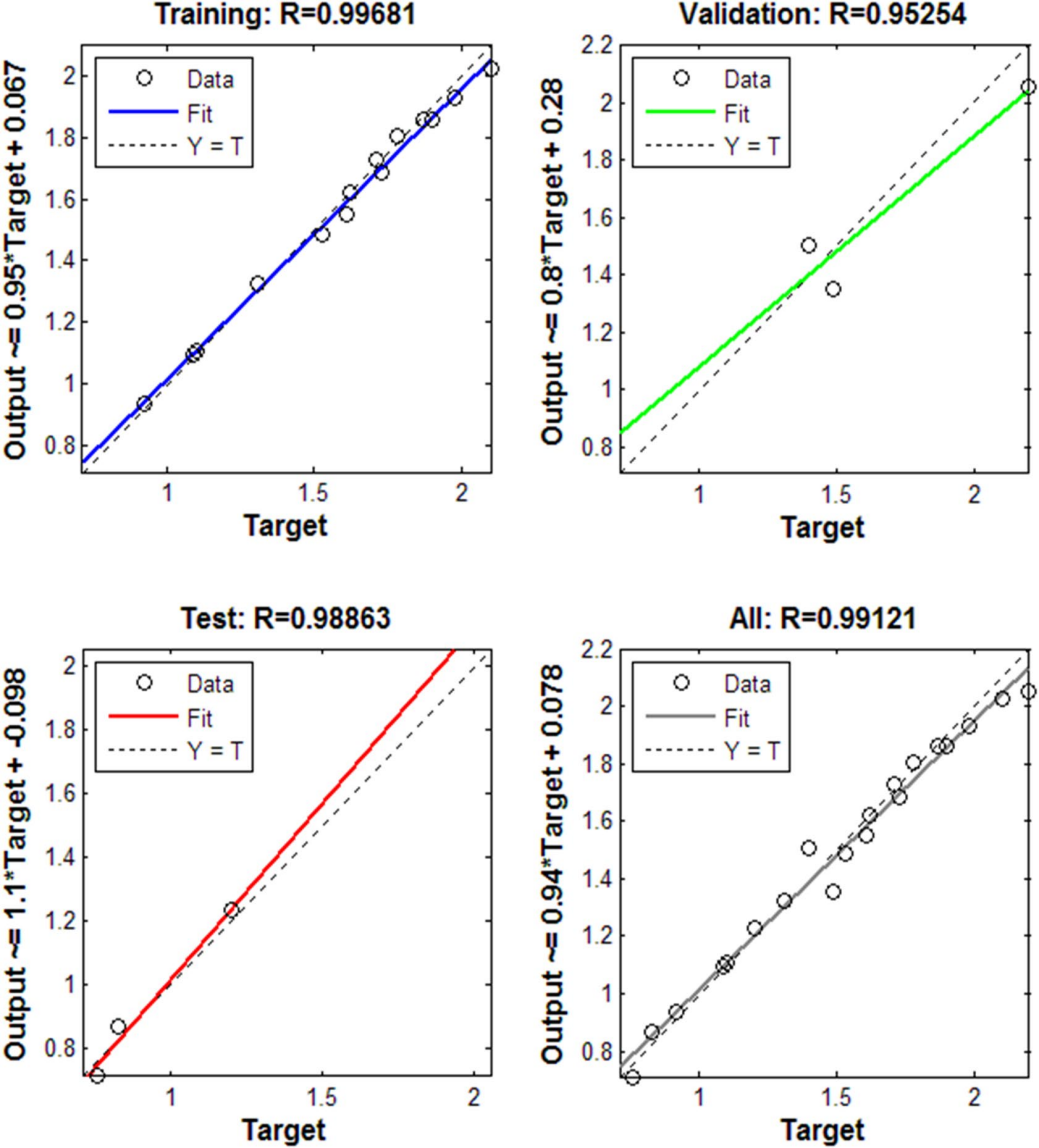
R^2^ values for training, testing, Validation, and all data for experimental and neural network predicted viscosity data.

In Fig. 15, the MSE for the three stages of training, testing, and Validation is shown as a function of the number of epochs. For the designed neural network, 70% of the input data were randomly assigned to training, 15% to testing, and the remaining to validation. As illustrated in Fig. 15, the MSE for all three phases decreases as the number of epochs increases. According to Figs. 11 and 15 epochs were used, and the network achieved the target in epoch 5, with an MSE of 0.017138. For testing data, increasing the number of epochs beyond 5 resulted in a higher MSE. Figure 16 shows the R^2^ values for all the divided parts of the dataset for the experimental and neural network–predicted viscosity data. The R^2^ for each category using the Purelin function is 0.99681, 0.95254, 0.98863, and 0.99121, respectively. Values closer to 1 indicate that the neural network with 7 neurons makes accurate predictions. Table 9 presents the weights and biases for the input, hidden, and output layers with 7 neurons, using the Purelin function. With 2 inputs and 7 neurons per input, a total of 7 weights were obtained. The biases for the first and second layers are 7 and 1, respectively.

**Table 9:** Weights and biases for input data, hidden layer, and output layer with 7 neurons using the Purelin function.

Purelin function				
Iw(1,1)			Iw(2,1)	
Number of neurons	Input 1	Input 2	[-0.0040479 -0.064179 -0.018435 -1.402 -0.19186 0.057877–0.24704]	
1	4.7028	2.4799	b(1)	b(2)
2	4.8772	2.7086	-4.1088	0.36735
3	2.0611	3.8624	-4.0098	
4	0.066198	-1.0772	-0.13611	
5	2.3555	3.0861	0.52729	
6	3.2944	1.4446	0.84927	
7	1.9606	2.8479	2.4294	
			3.9664	

The neural network, by learning its weights and biases, effectively models the complex relationships between input parameters (such as temperature and concentration) and the output (viscosity). While we did not perform an explicit, neuron-by-neuron analysis of individual weights, the overall behavior of the model and the influence of input parameters on the output reveal significant physical insights:

- **Effect of Temperature**: According to fundamental principles of thermodynamics and fluid mechanics, an increase in temperature increases the kinetic energy of molecules and nanoparticles. This facilitates their freer movement, thereby reducing resistance to flow (i.e., viscosity). Our model accurately captures this inverse relationship: as the input temperature increases, it predicts lower viscosity. This aligns perfectly with the observed physical behavior of most fluids.
- **Effect of Concentration**: Conversely, an increase in nanoparticle concentration implies a higher number of particles per unit volume. This leads to increased interactions between nanoparticles themselves and with the base fluid, potentially promoting particle aggregation and, subsequently, increasing flow resistance. Our model has also learned this direct relationship: as the input concentration increases, the predicted viscosity increases, consistent with experimental observations and existing theories of nanofluids.In essence, the neural network’s learned weights represent these fundamental physical relationships, illustrating how temperature and concentration.The high correlation coefficient of *R* = 0.98704 between the model’s predicted values and the experimental data indicates a very strong agreement. This high coefficient signifies that the neural network model has successfully learned the general trends and governing relationships within the dataset.The Mean Squared Error (MSE = 0.002188) is also relatively small, suggesting a high degree of accuracy in the model’s viscosity predictions. However, no model is perfect, and the presence of these minor errors can be attributed to several plausible physical factors:
- **Nanofluid Complexities**: The behavior of nanofluids can be intricate, influenced by factors such as nanoparticle size and shape, the type of base fluid, the presence of other additives, and phenomena like particle aggregation or sedimentation, which the model may not fully capture.
- **Experimental Conditions**: Minor errors might arise from subtle fluctuations in experimental conditions (e.g., slight variations in temperature or pressure) that affect the precise measurement of viscosity.
- **Model Limitations**: While powerful, neural networks might not be able to model all physical nuances across the entire data range perfectly.The strong agreement between the model’s predictions and experimental data (high R^2^) and the small errors demonstrate that the neural network has effectively learned and represented the dominant physical relationships governing temperature, concentration, and nanofluid viscosity.

Table 10 provides a comparative overview of recent studies on graphene oxide-based nanofluids, highlighting the reported increases in viscosity. The last row presents the results of this study, allowing direct comparison with previously published data on nanofluid type, concentration, temperature range, and observed viscosity changes. This comparison demonstrates the consistency and validity of the experimental and modeling approaches used in our work.

**Table 10:** Comparative summary of graphene oxide-based nanofluids: viscosity increases reported in recent studies and this work.

Researcher	Nanofluid type	Reported viscosity increase (%)
Kanti et al.^36^	GO/water (0–1 vol%,30–60 °C)	**≈ 177%** at 30 °C for pure GO
Huminic et al.^37^	GO-Si/water hybrid(0.4GO-0.6Si mixture, 20–50 °C)	For 0.4GO-0.6Si mixture: ≈ 179%-147% increase compared to the base fluid
Arachchi, et al.^38^	GO/water and prGO/water	Exact % not reported; focus mainly on thermal conductivity
Khouri et al.^39^	GO/water (0.01–0.1 wt%,40–85 °C)	Stated that viscosity increases with nanoparticle concentration, Nanofluid viscosity rose with increased GO nanoparticle concentration, reaching a minimum of 0.83 mPa·s at 85 °C and 0.01 wt %.
This study	GO/water (5–8 g/L, 20–35 °C)	(At 20 °C and a concentration of 5 g/L, the viscosity of the studied nanofluid was measured to be 1.4 cP. This represents a significant increase of approximately 16.67% compared to the viscosity of water 1.2 cP under similar conditions)

Kanti et al.^40^ investigated the synergistic effects of hybridizing SiO₂ and TiO₂ nanoparticles with graphene oxide in distilled water. Nanofluids were prepared at volume concentrations of 0.05–1% using PVP as a surfactant. The stability, viscosity, and thermal conductivity of the samples were evaluated at temperatures ranging from 30 to 60 °C, and characterization was performed using XRD, FESEM, DLS, and zeta potential analysis. Novel correlations were developed to estimate the thermophysical properties of the nanofluids. The highest viscosity increase was observed for the pure GO nanofluid. At 60 °C and 1% volume concentration, the thermal conductivity of the GO nanofluid was 14.4% and 9.8% higher than that of the GO-SiO₂ and GO-TiO₂ hybrid nanofluids, respectively. Based on performance enhancement ratios, hybrid nanofluids were identified as promising candidates for thermal applications above 45 °C, particularly in advanced cooling systems. The main objective of this study was to investigate the effects of concentration and temperature on the viscosity of graphene oxide nanofluids and to develop a predictive ANN model. Therefore, the paper focuses primarily on experimental data analysis and the development of an accurate predictive model. However, some fundamental chemical and physical characteristics of the nanofluids, such as the surface properties of graphene oxide, particle stability, and interactions between particles and the base fluid, were considered when selecting the concentration and temperature ranges and analyzing viscosity behavior. The surface nature of graphene oxide includes functional groups such as hydroxyl, epoxy, and carboxyl, which form hydrogen bonds with water molecules, creating a network of surface coverage around the particles that affects dispersion and viscosity. Particle stability prevents aggregation and sedimentation, and instability can lead to nonlinear behavior and fluctuations in viscosity measurements. Furthermore, interactions between particles and the base fluid, including electrostatic forces and hydrogen bonding, influence the nanofluid’s rheological behavior, leading to variations in flow resistance across different temperatures and concentrations. Therefore, even without conducting complex chemical and physical experiments, the analysis of viscosity data reflects the direct influence of these characteristics on nanofluid behavior. In summary, this study provides an experimental–analytical approach that can support.

## Conclusion

In our current study, the viscosity of graphene oxide/water NF was evaluated as a function of NF concentration and temperature. NF concentration from 5 to 8 g/L and temperature from 20 to 35 °C were chosen to report the following results:

- The viscosity of graphene oxide/water NF is affected by the changes in NF concentration and temperature. As the temperature increases, weaker van der Waals intermolecular forces reduce resistance to the mobility of adjacent NF layers. Therefore, NF viscosity decreases. The reverse results can be achieved if NF concentration increases^40^. This is because any increase in the concentration of nanographene oxide in the base fluid, Water, increases the impurity level of the base fluid.
- The proposed correlation, which is a function of temperature and concentration, satisfactorily predicts the experimental data, covering all viscosity data, with a regression value (R^2^ = 0.98704) and a mean square error value (MSE = 0.002188).
- The 2-7-1 neural network topology, using the Levenberg-Marquardt algorithm and the Purelin transfer function, achieved an R^2^ of 0.99122 and a mean squared error of 0.003712758, indicating reliable prediction of viscosity values.
- The proposed correlation and the experimental results are in good agreement, with a general overlap of the findings and a deviation of + 7.5% to -9.5% between the experimental and theoretical methods.
- For future research, it is recommended that the investigation of graphene oxide-based nanofluids be extended to higher concentrations and broader temperature ranges to better simulate industrial conditions. Hybrid nanofluids, which combine graphene oxide with metallic, ceramic, or polymer nanoparticles, could also be explored to enhance thermal conductivity, viscosity, and stability simultaneously. The effects of particle size, shape, and surface functionalization on fluid behavior should be systematically studied. Advanced predictive modeling, leveraging deep neural networks and machine learning algorithms, can yield more accurate property estimates and reduce the need for extensive experimental work. Computational fluid dynamics (CFD) simulations should be conducted to analyze flow behavior and temperature distribution in real equipment, complementing experimental findings. Long-term stability studies, including sedimentation, chemical degradation, and structural changes, are crucial for assessing the practical applicability. Environmental and toxicity assessments should be performed to evaluate ecological impacts before large-scale industrial applications. Economic analyses are necessary to examine production costs and potential benefits compared to conventional fluids. Furthermore, the application of nanofluids in emerging technologies such as microchannel cooling systems, solar energy devices, and advanced HVAC systems could be investigated. Ultimately, integrating experimental and theoretical studies will facilitate the development of comprehensive, reliable models to predict the performance of graphene oxide-based nanofluids under various conditions.

## Data Availability

The data that support the findings of this study are available from the corresponding author, upon reasonable request.

## List of symbols

ANN: Artificial neural network
MOD: Margin of deviation
XRD: X-ray diffraction
MSE: Mean squared error
RMSE: Root-mean-square deviation
MAE: Mean absolute error
R^2^: Coefficient of determination
FESEM: Field emission scanning electron microscopy

## Greek symbol

$$\varphi$$: Nanoparticle concentration
$$\mu$$: Viscosity

## Subscripts

bf: Base fluid
Corr: Correlation
Exp: Experimental
nf: Nanofluid
